# Fucoidan alginate and sulfated alginate microbeads induce distinct coagulation, inflammatory and fibrotic responses

**DOI:** 10.1016/j.mtbio.2025.101474

**Published:** 2025-01-10

**Authors:** Kalaiyarasi Vasuthas, Joachim Sebastian Kjesbu, Alessandro Brambilla, Maya Levitan, Abba Elizabeth Coron, Davi M. Fonseca, Berit L. Strand, Geir Slupphaug, Anne Mari A. Rokstad

**Affiliations:** a Centre of Molecular Inflammation Research (CEMIR), NTNU, Norway; b Department of Clinical and Molecular Medicine, NTNU, Norway; c Department of Biotechnology and Food Science, NTNU, Norway; d Clinic of Laboratory Medicine, St Olavs Hospital, Trondheim, Norway; e Proteomics and Modomics Experimental Core (PROMEC), NTNU and the Central Norway Health Authority, Norway

**Keywords:** Sulfated polysaccharides, Hydrogels, Surface, Fibrinolysis, ECM-Degrading proteases, LC/MS-Proteomics

## Abstract

This study investigates the host response to fucoidan alginate microbeads in comparison to sulfated alginate microbeads, which are relevant for immune protection in cell therapy. While sulfated alginate microbeads reduce fibrosis and inflammation, fucoidan, a kelp-derived polysaccharide rich in sulfate groups, has not been evaluated in this context. The study assesses surface reactivity to acute-phase proteins and cytokines using *ex vivo* human whole blood and plasma models. It also examines pericapsular overgrowth (PFO) in C57BL/6JRj mice, incorporating protein pattern mapping through LC-MS/MS proteomics.

Fucoidan alginate microbeads activated complement and coagulation, while both fucoidan and sulfated alginate microbeads induced plasmin activity. Fucoidan alginate microbeads exhibited a distinct cytokine profile, characterized by high levels of MCP-1, IL-8, IFN-γ, and reduced levels of RANTES, Eotaxin, PDGF-BB, TGF-β isoforms, along with higher PFO. The balance between plasmin activity and coagulation emerged as a potential predictor of fibrosis resistance, favouring sulfated alginate microbeads.

Explanted materials were enriched with both complement and coagulation activators (Complement C1q and C3, Factor 12, Kallikrein, HMW-kininogen) and inhibitors (C1-inhibitor, Factor H, Factor I). Fucoidan alginate microbeads predominantly enriched extracellular matrix factors (Fibrinogen, Collagen, TGF-β, Bmp), while sulfated alginate microbeads favoured ECM-degrading proteases (Metalloproteases and Cathepsins).

This study reveals significant differences in host responses to fucoidan and sulfated alginate in microbeads. The plasmin activity to coagulation ratio is highlighted as a key indicator of fibrosis resistance. Additionally, the preferential enrichment of ECM-degrading proteases on the material surface post-implantation proved to be another crucial factor.

## Highlights

• Fucoidan alginate and sulfated alginate microbeads show distinct surface properties.
• Different complement activation, coagulation and plasmin activity profiles.
• Different enrichment of inhibitors and activators of complement, coagulation and fibrinolysis.
• Difference in pro-fibrotic proteins versus ECM-degrading proteases related to fibrosis.
• Ratio plasmin activity to coagulation predicts the material's potential to resist fibrosis.

## Graphical abstract

**Figure:**
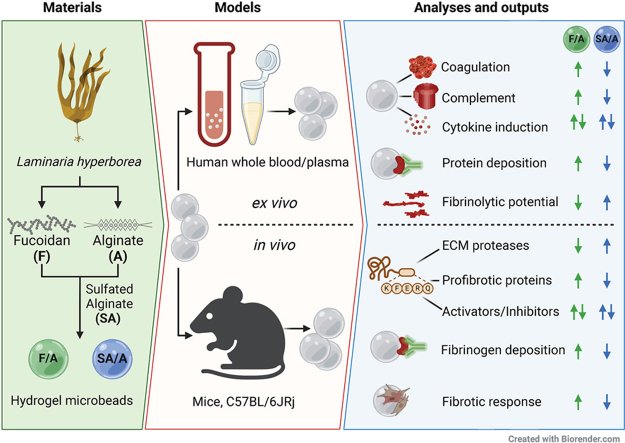

## 1 Introduction

Fucoidan, a sulfated and branched polysaccharide derived from kelp, exhibits structural variability depending on the kelp species [[1], [2], [3]]. Fucoidan from *Laminaria hyperborea* primarily consists of fucose, with a flexible backbone of (1 → 3)-α-l-fucopyranose, and other glycosidic linkages including 1 → 2 and 1 → 4, short side chains, smaller fractions of galactose residues, and a high degree of sulfation [1,4]. In recent years, fucoidans have attracted substantial research interest due to their wide range of biological activities, highlighting their potential for therapeutic applications [5,6]. These activities include anti-cancer properties [7] and context-dependent pro-, or anti-inflammatory effects [3]. Moreover, fucoidans are recognized as immunomodulatory agents. Some studies report their ability to activate monocytes and lymphocytes, while others demonstrate their role in inhibiting leukocyte migration and recruitment [8,9]. Previously, we demonstrated that the bioactivity of *L. hyperborea-*derived fucoidan in human whole blood, assessed through various cytokines, growth factors, coagulation and complement activation, depends on factors such as molecular weight, degree of sulfation and polymer concentration [4]. Collectively, the literature highlights the diverse bioactivities of fucoidan; however, discrepancies in reported results may stem from its source-dependent structural variations, purity, mode of presentation, and the specific experimental conditions in which the fucoidans are applied [3,10,11].

Alginates, another group of polysaccharides from kelp, are commonly used in tissue engineering and cell encapsulation, due to their capacity to form ionically crosslinked hydrogels under cell-compatible conditions [12]. Alginates are linear copolymers of (1 → 4)-linked β-d-mannuronic (M) acid and α-l-guluronic acid (G) residues [13]. Alginate hydrogel microbeads containing islets or hepatocytes have been implanted in humans for the treatment of type 1 diabetes or acute liver failure, demonstrating good biocompatibility and safety. However, their long-term therapeutic efficacy is limited by fibrotic responses [14,15]. Alginate can be chemically modified with sulfate groups, modulating its bioactive properties both in solution and in mixed gels with unmodified alginate [16,17]. Sulfated alginate (SA) microbeads have shown reduced inflammatory potential in human whole blood [18] and decreased pericapsular fibrotic overgrowth (PFO) in C57BL/6 mice [16]. The addition of sulfate groups to alginate promotes the enrichment of inhibitory factors within the complement and coagulation systems, as well as fibrinolytic factors altering its bioactivity [19].

Unlike alginate, fucoidan lacks an inherent crosslinking mechanism. Accordingly, most research on fucoidan bioactivity utilizes solutions of fucoidan or explores covalent crosslinking strategies involving chemically modified functional groups [20] or crosslinking with macromolecules bearing cationic functional groups [21]. Fucoidan has also been incorporated into composite hydrogels, where the crosslinking is provided by another component such as agarose or alginate [22]. These composite hydrogels have been investigated for use in cell encapsulation for the treatment of type 1 diabetes [22,23]. However, the immunogenic properties of fucoidan in the hydrogel state remain largely unexplored, which is crucial for understanding its functional outcomes in transplantation and tissue engineering applications.

This study aimed to determine the host responses of the naturally sulfated polysaccharide fucoidan when embedded within alginate hydrogel microbeads. These were compared to microbeads containing sulfated alginate, previously shown to yield promisingly low inflammatory and fibrotic responses. Previously, we demonstrated that using a combination of an *ex vivo* human whole blood model for initial inflammatory responses and an *in vivo* C57BL/6 mouse model to assess pericapsular overgrowth was effective in revealing key characteristics of material host responses crucial for long-term performance [16]. Additionally, we have shown that proteomics can serve as a valuable tool to detail protein absorption and binding patterns to key proteins involved in inflammation [19]. Here we included a proteomics approach to explanted microspheres following mice transplantation together with the assessment of PFO, combined with analysis of initial inflammatory responses in human blood.

This work provides detailed analysis of the host responses elicited by fucoidan and sulfated alginate containing microspheres highlighting their unique biological surface properties. The type of sulfated polysaccharide in the microbeads, resulted in variation in pericapsular overgrowth and cytokine induction elicited by the microbeads. Differences also include complement, and coagulation profiles, with varying enrichment of their inhibitors and activators. In summary, the fucoidan alginate microbeads were more proinflammatory and pro-fibrotic when compared to sulfated alginate microbeads. Our results suggest that the ratio of plasmin activity to coagulation could serve as a predictor of a material's potential to resist fibrosis. Also, the presence of pro-fibrotic proteins versus extracellular matrix-degrading proteases on the material surfaces could be relevant for fibrosis.

## 2 Materials and methods

### 2.1 Polysaccharides

Ultrapure (UP) alginates (A) were obtained from Novamatrix (Sandvika, Norway). The unmodified alginate used in the microbeads containing sulfated alginate (SA) or fucoidan (F) was a low viscosity high G (LVG) alginate (68 % G, 237 kDa). The substrate alginate for sulfation was a medium viscosity high G (MVG) alginate (66 % G, 233 kDa). Sulfation was performed as described by Arlov et al. [24], resulting in a degree of sulfation (DS) of 0.83 and a weight average molecular weight (M_w_) of 162 kDa. The G content of alginates was determined by ^1^H NMR based on the methods established by Grasdalen et al. [25,26]. Molecular weight was determined using SEC MALLS [27]. The fucoidan was extracted from *Laminaria hyperborea* by International Flavors & Fragrances (IFF), formerly FMC Biopolymer (Sandvika, Norway), and subsequently purified under GMP conditions by NovaMatrix (Sandvika, Norway). The same batch of fucoidan was structurally characterized in a previous study by Kopplin et al., showing a composition of 97.8 % fucose and 2.2 % galactose, with an M_w_ 469 kDa and a DS of 1.7. Sulfate groups were located axially at 4C- and equatorially at 2C positions. Glycosidic linkage analyses by Kopplin et al., [4] revealed a highly branched (22.4 %) structure consisting of (1 → 3)-α-l-fucopyranose (31.9 %), and 1→2-linked (13.2 %) and 1→4-linked (7.7 %) fucopyranose.

### 2.2 Preparation of microspheres

Microspheres were produced with an in-house built (NTNU, Trondheim) electrostatic droplet generator system operated at 6–7 kV with a 0.35 mm nozzle, and a syringe pump (Cole-Parmer Instrument Company LLC, Vernon Hills, IL, USA) for alginate extrusion at a flow rate of 10 mL/h [28]. Solutions were prepared in conditions compatible with cell encapsulation, using sterile, hypotonic, nonpyrogenic water (B. Braun Melsungen AG, Melsungen, Germany). Alginate, sulfated alginate and fucoidan were dissolved with 0.3 M mannitol (VWR International BVBA, Leuven, Belgium) for physiological osmolarity, and 10 mM HEPES (PanReac AppliChem GmbH, Darmstadt, Germany) at pH 7.3–7.4, and sterile filtered. For crosslinking, a solution of 50 mM CaCl_2_ (Sigma-Aldrich, St. Louis, MO, USA) with 1 mM BaCl_2_ (Merck KGaA, Darmstadt, Germany), 0.15 mM mannitol and 10 mM HEPES at pH 7.3–7.4 was used. Following gelation, the crosslinking solution was removed, and microspheres were rinsed and stored in a physiologically compatible washing solution containing 0.9 % NaCl (VWR International BVBA, Leuven, Belgium), 2 mM CaCl_2_ and 10 mM HEPES at pH 7.2–7.4. Alginate-poly-L-lysine (AP) microcapsules were produced as positive controls for complement activation and cytokine responses in the human whole blood model [29]. AP microcapsules were prepared by gelling alginate microbeads in 50 mM CaCl_2,_ followed by a 10 min incubation in 0.1 % poly-L-lysine (PLL) (Sigma-Aldrich, St, Louis, MO, USA) to form an outer PLL coating. The mean diameters of the microspheres, ranging from approximately 370 μm–550 μm (±3 %–14 %, n = 30), were measured using brightfield microscopy (LSM800, Carl Zeiss MicroImaging GmbH, Göttingen, Germany), and analyzed with Image J software v. 1.54f, (National Institutes of Health, New York, USA) using an EC Plan-Neofluar 2.5 × /0.085 M27 objective. Microbeads were produced with total polymer concentrations ranging from 1.80 % to 2.16 % (w/v) and are named based on the fractions of fucoidan (F; 10–40 %) and sulfated alginate (SA; 10–40 %) and hence named F/A 10/90 - F/A 40/60 and SA/A 10/90 - SA/A 40/60. These ratios were selected based on methodologies employed by past work to facilitate droplet generation of microspheres with appropriate stability [16,18,30]. Furthermore, these conditions allowed for matched concentrations or mean sulfate group content in select microbeads. Table 1 provides an overview of the polymer concentrations (% w/v) and their corresponding names and sulfate groups in the microbeads.

**Table 1:** **Microspheres used within this study.** Provided are the names and polymer constituents of alginate (A), fucoidan (F) and sulfated alginate (SA) in percent weight by volume.

**Microspheres**	Alginate (**A**)	Fucoidan (**F**)	Sulfated alginate (**SA**)	Poly-L-lysine (**PLL**)	SO_3_^-^/monomer
**F/A 10/90**	1.62	0.18			0.17
**F/A 17/83**	1.80	0.36			0.28
**F/A 20/80**	1.44	0.36			0.34
**F/A 40/60**	1.08	0.72			0.68
**SA/A 10/90**	1.62		0.18		0.08
**SA/A 20/80**	1.44		0.36		0.17
**SA/A 40/60**	1.08		0.72		0.33
**A**	1.80				
**AP**	1.80			0.10	

### 2.3 Human whole blood model

The human whole blood model developed by Mollnes et al. [31], later adapted to assess microspheres [32,33], is commonly used to measure the initial inflammatory properties, including complement and coagulation activation. The original protocol employed the thrombin inhibitor lepirudin as an anticoagulant, whereas in this study we used hirudin as a thrombin inhibitor by drawing blood into hirudin-containing (>525 ATU/mL) vials (S-Monovette®, Sarstedt AG & Co., Numbrecht, Germany). Microspheres (50 μL) were aliquoted in low-activating polypropylene vials (Cat:366656, 1,8 mL: Nunc, Roskilde, Denmark) in 0.9 % NaCl (50 μL, B. Barun, Melsungen, Germany) for a total volume of 100 μL. PBS with CaCl_2_ and MgCl_2_ (Cat: D8662, Sigma Aldrich) was added (100 μL), bringing the total volume to 200 μL. Zymosan (10 μg/vial in 0.9 % NaCl, Sigma Aldrich, St. Louis, MO, USA, Z4250) and glass tubes (Cat: 367614, BD Vacutainer) were included as positive controls for complement/cytokine and coagulation activation, respectively. Whole blood from healthy volunteers (N = 11) was drawn into hirudin-containing vials. 500 μL of blood was added to the microbeads and controls, followed by incubation at 37 °C for either 40 or 240 min under continuous rotation. Complement and coagulation activation were stopped by adding EDTA (Sigma Aldrich Corp, St. Louis, MO, USA) to a final concentration of 10 mM. Plasma samples from each condition were collected and aliquoted following centrifugation at 3000*g* for 15 min. The samples were stored at −20 °C for further analysis.

For a subset of studies, hirudin anticoagulated plasma was used instead of human whole blood. In these cases, plasma was prepared by centrifuging hirudin-anticoagulated blood at 3000 g for 15 min and was used either fresh or after freezing at −80 °C.

### 2.4 Fluid phase TCC quantification

Activation of the complement cascade was assessed by quantifying the fluid phase terminal complement complex (TCC) in the plasma using a sandwich enzyme-linked immunosorbent assay kit (ELISA; Hycult Biotech, HK328) according to the manufacturer's instructions.

### 2.5 Prothrombin fragment F 1 + 2 (PTF1.2) quantification

The concentration of prothrombin fragment F1+2 (PTF1.2) was measured as an indicator of coagulation activation, using the Enzygnost F1+2 monoclonal ELISA kit (Siemens Healthcare Diagnostics, Marburg, Germany). The kit was used according to the manufacturer's protocol, with dilution adjustments ranging from 10 × and 1000 × , depending on the stimuli.

### 2.6 Profiling of cytokines, chemokines, and growth factors by multiplex assay

The concentrations of plasma cytokines were measured using a multiplex assay (Bio-Plex Human cytokine 27-Plex Panel, Bio-Rad Laboratories, Hercules, CA). The panel of analytes included interleukins: IL-1 beta (IL-1β), IL-1 receptor antagonist (IL-1ra), IL-2, IL-4, IL-5, IL-6, IL-7, IL-8 (CXCL8), IL-9, IL-10, IL-12, IL-13, IL-15, IL-17; chemokines: eotaxin (CCL11), chemokine (C-X-C motif) ligand 10 (IP-10/CXCL10), monocyte chemoattractant protein-1 (MCP-1/CCL2), macrophage inflammatory protein-1-alfa (MIP-1α/CCL3), macrophage inflammatory protein-1-beta (MIP-1β or CCL4), RANTES (regulated upon activation, normal T cell expressed and presumably secreted, CCL5); growth factors: basic fibroblast growth factor (b-FGF), granulocyte colony-stimulating factor (G-CSF), granulocyte-macrophage colony-stimulating factor (GM-CSF), platelet-derived growth factor-BB (PDGF-BB), vascular endothelial growth factor (VEGF), transforming growth factor beta isoforms (TGF-β1, TGF-β2, TGF-β3), interferon-gamma (IFN-γ) and tumor necrosis factor-alpha (TNF-α). The multiplex assay was performed according to the manufacturer's instructions, using half the recommended number of beads.

### 2.7 Surface plasmin activity potentials

Microspheres were pretreated with hirudin-anticoagulated plasma for 4 h at 37 °C and subsequently washed three times with 0.9 % NaCl. Plasmin assay buffer (PAB) (final volume 80 μL) provided with the Plasmin Activity Assay Kit (Abcam, Colorimetric, ab273301 or Fluorometric ab204728) was added to the retrieved microspheres (50 μL). 10 μL diluted positive control (provided with the kit) was mixed with 70 μL of PAB. The negative control was prepared according to the instructions provided by the manufacturer by mixing 20 μL of diluted plasmin inhibition mix with 60 μL of PAB. Samples and controls were incubated at 37 °C in the dark for 30 min, followed by the addition of 20 μL of reaction buffer (provided with the kit). Active plasmin present in the samples hydrolyzes the synthetic substrate, releasing the chromophore *p*NA, which was measured at 450 nm at various time points (T0, 30 min, 60 min, 4 h and 24 h). The colorimetric signal is directly proportional to plasmin activity. To further investigate the functional predictability of materials in terms of fibrosis and fibrinolysis, the ratios between *Δ*_*PA*_ (change in plasmin activity) to *Δ*_*PTF 1.2*_ (change in PTF1.2 amount) at different time points were calculated (Eq. (1)). Where the ratio at time T,

(1) RatioT=ΔPAT/ΔPTF1.2T

$$\Delta_{PA}T=\left(T_{PA}-{T0}_{PA}\right)\;;\;\Delta_{PTF1.2}T=\left(T_{PTF1.2}-{T0}_{PTF1.2}\right)$$

### 2.8 Visualization of acute phase activators and inhibitors by CLSM

For each sample, microspheres (50 μL) in saline (50 μL) were incubated with human lepirudin-plasma (300 μL) for 24 h at 37 °C under constant rotation. The microspheres were washed in PBS before incubation with antibodies. The subsequent incubation steps lasted 30 min and were carried out at 37 °C with constant rotation. C3c was assessed by incubating the microspheres with FITC-conjugated polyclonal rabbit anti-human C3c (50 μg/mL) (DAKO F0201, Dako Cytomation, Glostrup, Denmark) or its control, FITC-conjugated polyclonal swine anti-rabbit Ig (50 μg/mL) (Dako Cytomation, Glostrup, Denmark). C1q and C1 inhibitor were evaluated by incubation of the microspheres with rabbit anti-human C1inh (1:150) (Nordic BioSite, Täby, Sweden), followed by secondary Alexa Fluor 647-conjugated goat anti-rabbit IgG (1:1500) (Invitrogen, Eugene, Oregon), followed by either FITC-conjugated rabbit anti-human C1q (50 μg/mL) (Dako Cytomation, Glostrup, Denmark) or its control, FITC-conjugated swine anti-rabbit Ig (50 μg/mL) (Dako Cytomation, Glostrup, Denmark). Factor H (FH) was assessed by incubating the microspheres with polyclonal sheep anti-human FH (50 μg/mL) (PC030, The Binding Site, Birmingham, UK), followed by secondary CF633-conjugated donkey anti-sheep IgG (10 μg/mL) (SAB4600134, Sigma Aldrich). Plasminogen/plasmin was evaluated by incubation of the microspheres with polyclonal sheep anti-human plasminogen (50 μg/mL) (The Binding Site, Birmingham, UK), followed by secondary CF633-conjugated donkey anti-sheep IgG (20 μg/mL) (Sigma Aldrich). Factor 12 (FXII/FXIIa) was evaluated by incubation of the microspheres with polyclonal sheep anti-human FXII (Nordic Diagnostica Service AB, HTI, Kungsbacka, Sweden, 50 μg/mL), followed by secondary CF633-conjugated donkey anti-sheep IgG (Sigma-Aldrich, 20 μg/mL). As a control for FH, plasminogen/plasmin, and FXII/FXIIa, staining was performed using only the secondary antibody, CF633-conjugated donkey anti-sheep IgG (20 μg/mL).

The microspheres were washed with 0.9 % NaCl with 2 mM CaCl_2_ before deposition was visualized using a Zeiss LSM 510 confocal microscope with 488 nm argon- and 633 nm helium-neon lasers (Carl Zeiss MicroImaging GmbH, Göttingen, Germany). Optical cross-sections (pinhole: 21.5 μm) were captured through the equator of the microspheres using laser scanning and differential interference contrast. 3D projections through the microspheres were created from z-stacks using Image J software (V1.54i) (National Institutes of Health, Bethesda MD, USA). CLSM settings were consistent across all images. Intensity quantification of each staining was performed using Image J with normalized quantification performed using 9–12 microspheres per condition.

To determine if FH binding was secondary to complement C3 activation, C3 was inhibited prior to FH quantification by CLSM imaging. The complement inhibitor used was CP40, a compstatin analogue (sequence: 14-aa cyclic peptide [D]Tyr-Ile-[Cys-Val-Trp(Me)-Gln-Asp-Trp-Sar-Ala-His-Arg-Cys]-mIle-NH2) kindly provided by J. D. Lambris [34]. CP40 was mixed with plasma (20 μM) for 10 min before adding the microspheres. The microspheres were then incubated overnight with either CP40-treated or nontreated plasma, stained with sheep anti-human FH or secondary antibody only, and visualized by CLSM as detailed above.

### 2.9 Animals and surgical procedures

Immunocompetent C57BL/6JRj mice were obtained from Janvier Labs (Saint Berthevin Cedex, France). All mice were female, with an average age of 10 weeks at the start of the experiment and with body weights ranging from 18.0 g to 23.5 g. Each mouse was housed individually with provided nesting materials, and their health and well-being were monitored throughout the study. Following disinfection with ethanol, a small incision was made through the skin to allow for the intraperitoneal implantation of microspheres (∼0.5 mL) using a catheter. The total injection volume, including the washing solution, was approximately 1 mL. General anaesthesia was induced using 1.5–3.0 % isoflurane (Baxter International Inc., Deerfield, IL, USA), and local analgesia was provided with subcutaneous injections of 40–50 μL (0.5 mg/mL) of Marcaine (AstraZeneca plc, Cambridge, UK) was given subcutaneously for local analgesia. Microspheres were explanted after 14 days. Mice were first anesthetized, followed by euthanasia through cervical dislocation. Microspheres were retrieved by lavage with 20 mL of washing solution.

### 2.10 Assessment of fibrosis and fibrinogen after *in vivo* deposition on microspheres

Microspheres, explanted from mice after 14 days were imaged using a Zeiss LSM800 CLSM equipped with an EC Plan-Neofluar 2.5 × /0.085 M27 objective. Microspheres (N = 100) were assessed for PFO and categorized into intervals of 0–25 %, 25–50 %, 50–75 % and 75–100 %. Fibrinogen deposition was evaluated on microspheres free of PFO by CLSM. Alginate microspheres were stained with FITC-conjugated polyclonal goat anti-mouse fibrinogen (Nordic-MU-bio, Susteren, Netherlands). As a control for non-specific binding, FITC-conjugated goat isotype control IgG (Nordic-MU-bio, Susteren, Netherlands) was used. For visualization of cell adsorption, DRAQ5™ (BioLegend Europe BV, London, UK, 1:1000) was used. A C-Apochromat 10 × /0.45 W objective was used for imaging of fibrinogen staining, with lasers for FITC-labelled antibodies (diode laser 488 nm, with detection wavelength 410–617 nm), and DRAQ5™ staining (diode laser 640 nm, with detection wavelength 650–700 nm). Optical cross-sections of 30 μm through the equator of the microspheres were captured. 3D projections were generated from z-stacks using ImageJ software v. 2.1.0/1.53c (National Institutes of Health, New York, USA).

### 2.11 LC-MS/MS proteomic analysis

The AP, SA/A 20/80, and F/A 20/80 microspheres were retrieved from mice 24 h post-implantation and washed three times for 5 min in 0.5 mL 0.9 % NaCl on a rotary shaker (400 rpm) at room temperature. After an additional washing with 200 μL tris-HCl (50 mM, pH 8.0), the microspheres were incubated in 100 μL of reduction and alkylation buffer (10 mM tris (2-carboxyethyl) phosphine, 40 mM chloroacetamine, 100 mM tris-HCl pH 8.0) for 60 min at 50 °C. Trypsin (1.2 μg, Thermo Fisher Scientific) was then added, and the microspheres were incubated overnight on a shaker at 37 °C, 400 rpm). For peptide desalting, C18 stage tip columns were washed with 50 μL of methanol and 50 μL of acetonitrile, followed by a 2-min centrifugation at 1500×*g*, and equilibrated with ammonium bicarbonate (10 mM). The samples were initially centrifuged at 16000×*g* for 10 min, and the supernatants were added to the columns and centrifuged for 2 min at 1500×*g*. Flow-through was reloaded to the stage tips and centrifuged for 1 min at 1500×*g*. The stage tips were washed two times with 60 μL of ammonium bicarbonate (10 mM) and centrifugated for 1 min at 1500×*g*. Peptides were eluted twice with 60 μL of acetonitrile (70 %), and ammonium bicarbonate (10 mM), centrifuged for 1 min at 1500×*g* for each elution, and the combined eluates evaporated to dryness. Dried peptides were reconstituted in 60 μL of formic acid (0.1 %), vortexed, and agitated at 4 °C for 3 h at 900 rpm. The samples were then centrifuged at 16000×*g* for 15 min and the supernatants were transferred to MS vials for LC-MS/MS analysis. LC-MS/MS analysis was performed on a timsTOF Pro 2 (Bruker Daltonics) connected to a nanoElute (Bruker Daltonics) HPLC. Peptides were separated on an Aurora Ultimate (75 μm × 25 cm, IonOpticks) column with a CaptivSpray insert and kept at 50 °C. LC employed running buffers A (0.1 % formic acid) and B (0.1 % formic acid in acetonitrile) with a gradient from 2 % to 40 % B for 40 min at 250 nL/min, then 40 %–95 % B for 10 min at 400 nL/min, where it was kept for 10 min. The MS instrument was operated in DDA PASEF mode with 10 PASEF scans per acquisition cycle and accumulation and ramp times of 100 ms each. The ‘target value’ was set to 20,000 and dynamic exclusion was activated and set to 0.4 min. The quadrupole isolation width was set to 2 Th for m/z < 700 and 3 Th for m/z > 800.

MaxQuant [35] version 2.4.9.0 was used to perform a database search of the spectra against the Mouse reference proteome including isoforms and unreviewed proteins (downloaded 2023-08-18 from UniProt (https://www.uniprot.org/). Search parameters included trypsin as the protease with a maximum of 2 missed cleavages, deamidation of asparagine/glutamine, oxidation of methionine and N-terminal acetylation as variable modifications, with carbamidomethylation of cysteine as fixed modification. Up to 5 modifications per peptide were allowed. LC-MS RunType was TIMS-DDA with Match Between Runs enabled. Reverse decoys were used to control for false discovery rate (FDR), set at 1 % for both peptide and protein levels. Label-free quantification was conducted by using the MaxLFQ algorithm [36], with only unique peptides (modified and unmodified) included in the analysis. The mass spectrometry proteomics data have been deposited to the ProteomeXchange Consortium via the PRIDE [37] partner repository with the dataset identifier PXD055860.

### 2.12 Statistical methods

Statistical comparisons between fucoidan alginate microbeads, other microbead types, and controls were performed by Repeated measures (RM) one-way ANOVA with Geisser-Greenhous corrections and Tukey's multiple comparisons test. Due to the limited sample size (N = 3–11), data were assumed to have non-normal distributions and were log-transformed prior to analysis. All statistical analyses were performed using Graph Pad Prism v10.2.1.

### 2.13 Ethics

All human blood-based experiments were compliant with the ethical approval granted by the Regional Ethic Committee at the 10.13039/100009123Norwegian University of Science and Technology under the approval number 2009/2245. Animal experiments were conducted in accordance with the national guidelines governing the care and use of laboratory animals and were approved by the Norwegian Food Safety Authority (application ID number 23927).

## 3 Results

### 3.1 Fucoidan alginate microbeads activate complement while also binding complement inhibitors

**Fig. 1:**
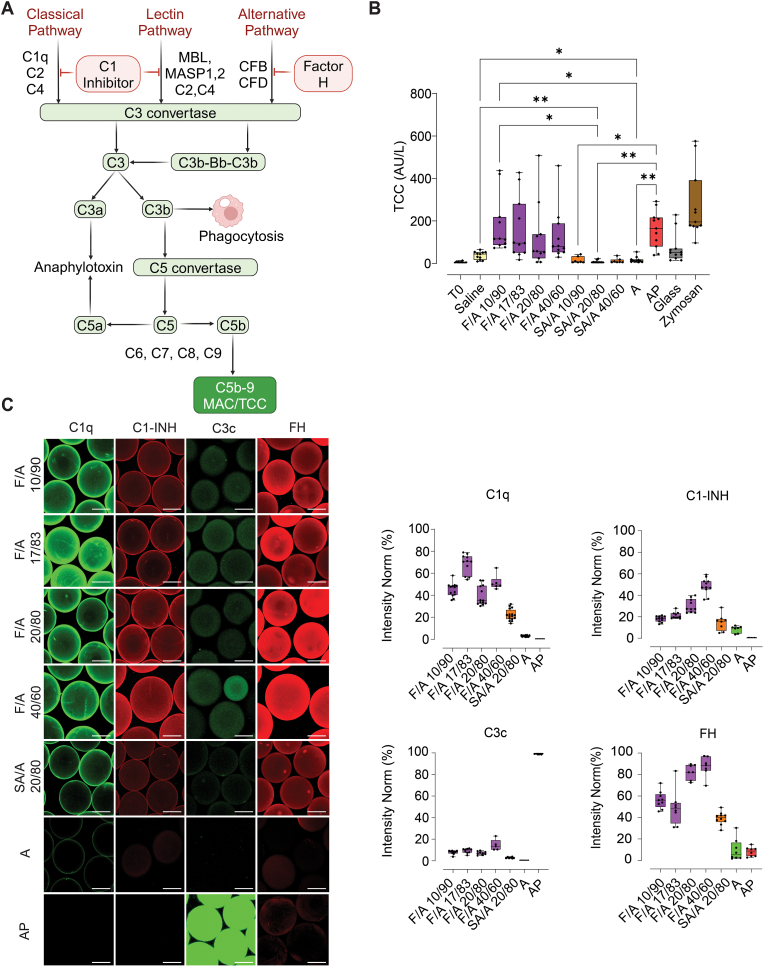
Fucoidan alginate microbeads induce complement and enrich for complement activators and inhibitors. **(A)** Illustrative diagram of the complement pathway (Created with BioRender.com). **(B)** Terminal complement complex (TCC, sC5b-9) was quantified after 240 min of incubation in hirudin anticoagulated human whole blood (N = 11 donors). Values are given as mean with 95 % CI and significance as p ≤ 0.05(∗), p ≤ 0.01(∗∗), p ≤ 0.001(∗∗∗), p ≤ 0.0001(∗∗∗∗) of the different fucoidan alginate microbeads (F/A), compared to control microspheres; SA/A, A and AP **(C)** Visualization of complement activators and inhibitors, C1q, C1-INH, C3c and Factor H, by CLSM after 24h incubation in hirudin-plasma including intensity quantification (N = 6–12 microspheres from two experiments). Scale bar: 200 μm.

The terminal complement complex (TCC) serves as a hallmark of overall complement activation. All fucoidan-alginate microbeads (F/A 10/90, F/A 17/83, F/A 20/80, F/A 40/60) increased TCC levels compared to baseline (T0), saline control, alginate (A) and sulfated alginate (SA/A) microbeads, reaching levels comparable to the AP positive control microcapsule. Specifically, F/A 10/90 induced a significant increase in TCC compared to SA/A 20/80 (p ≤ 0.05). SA/A and alginate (A) exhibited the lowest potential for complement activation. No significant differences in TCC levels were observed among the four fucoidan alginate microbead formulations (Fig. 1B). Similarly, no significant differences in TCC levels were found among the three blends of sulfated alginate microbeads (SA/A 10/90, SA/A 20/80, SA/A 40/60) (Fig. 1B). AP microcapsules induced significantly elevated TCC levels compared to SA/A and A but not compared to F/A microbeads.

Visualization and intensity quantification of the complement activators C1q and C3, and their inhibitors, C1 esterase inhibitor (C1-INH) and factor H (FH) are shown in Fig. 1C. The results clearly demonstrate the enrichment of C1q, C1-INH and FH on the F/A microbeads. The C1 complex comprises subcomponents C1q, C1r, and C1s, where the plasma protein C1-INH covalently binds to active C1r and C1s to irreversibly inactivate the C1 complex. Enrichment of both C1q and C1-INH was higher on the F/A microbeads compared to SA/A, A and AP (Fig. 1C). Notably, C1-INH binding increased with the concentration of fucoidan in the microbeads. Some enrichment of C1q and C1-INH was also found on the SA/A compared to A and AP.

A critical step in the complement pathway involves the proteolytic cleavage of C3, leading to the formation of C3 convertase (C3bBb), which is regulated by Factor H. Factor H inhibits the formation of both C3 and C5 convertases by promoting the dissociation of Bb from C3b, and subsequent C3c formation [38]. C3c, the largest subunit of C3, was strongly enriched on AP microcapsules, weakly enriched on FA/A and SA/A, and absent on A microbeads. This was consistent with the prominent binding of FH on the F/A microbeads, which was linked with the concentration of fucoidan. SA/A microbeads also exhibited elevated FH binding but albeit to a lesser extent than the F/A microbeads (Fig. 1C and Supplementary Fig. S1).

### 3.2 Factor H binds to fucoidan and sulfated alginate microbeads, regardless of activated C3

**Fig. 2:**
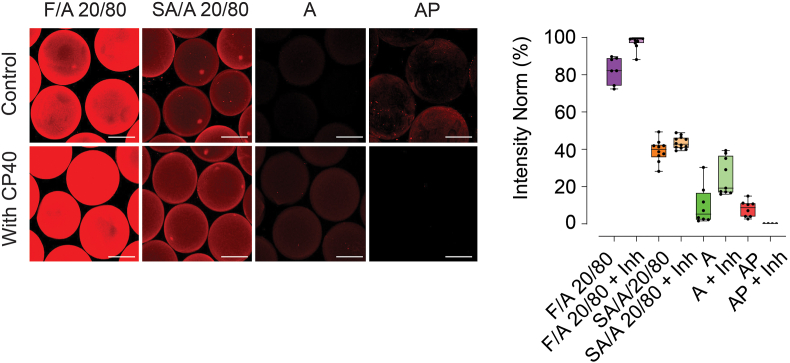
Factor H enrichment on fucoidan alginate microbeads and control microspheres with and without C3 inhibition. Visualization of Factor H by CLSM after staining with polyclonal sheep anti-human factor H and secondary staining with CF633-conjugated donkey anti-sheep IgG with intensity quantification (mean value of 6–12 microspheres in each condition). C3 inhibitor Compstatin; CP40 [39]. Scale bar: 200 μm.

The binding of FH to the microsphere surfaces may occur through direct interaction with polysaccharides or as a secondary event following cleavage of C3 into C3b/iC3b/C3dg [38]. To elucidate whether FH enrichment was the result of a direct interaction or secondary binding to activated C3, we inhibited C3 activation using compstatin CP40 [39] and visualized FH enrichment by CLSM (Fig. 2). Stained images and their intensity quantifications demonstrate that the binding of FH on the F/A and SA/A microbeads was not dependent on C3 activation. Intriguingly, inhibition of C3 by CP40 tended to enhance the FH binding on the F/A 20/80, SA/A 20/80 and A. In contrast, the AP microcapsules showed reduced FH upon inhibition of C3 (Fig. 2). Cumulatively, these findings confirm that FH binding to F/A and SA/A microbeads is inherent and independent of C3 activation, whereas binding to AP microcapsules occurs as a secondary event mediated by activated C3. In addition, cross-sectional images (2D) reveal that FH can penetrate the fucoidan- and sulfated alginate-containing microbeads with permeation increasing upon C3 inhibition (Supplementary Fig. S2).

### 3.3 Fucoidan alginate microbeads activate coagulation and trigger plasmin activity

**Fig. 3:**
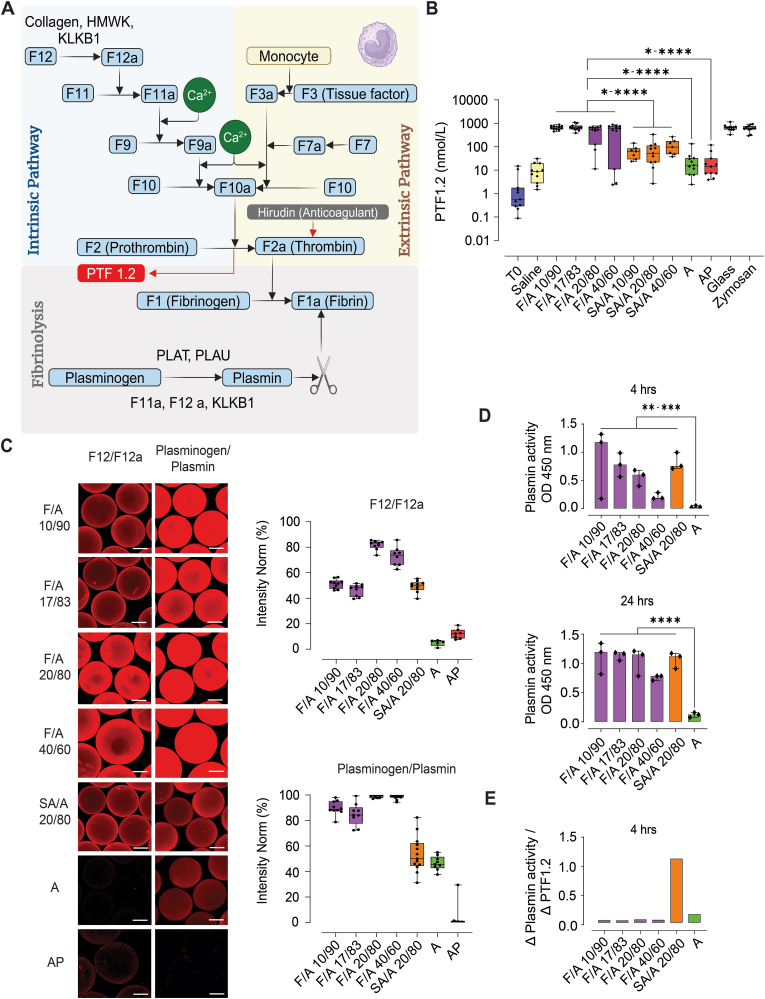
Fucoidan alginate microbeads potential to induce coagulation activation and plasmin activity. **(A)** Illustrative diagram of the coagulation cascade and plasmin-plasminogen system (Created with BioRender.com). **(B)** Quantification of prothrombin factor 1.2 (PTF1.2) after 240 min incubation in hirudin anticoagulated human whole blood (N = 11donors). Values are given as mean with 95 % CI and significance as p ≤ 0.05(∗), p ≤ 0.01(∗∗), p ≤ 0.001(∗∗∗), p ≤ 0.0001(∗∗∗∗) from F/A microbeads of various compositions compared to control microspheres; SA/A, A and AP. **(C)** Visualization of coagulation proteins; F12/F12a and Plasminogen/plasmin in human plasma by CLSM after 24 h incubation with intensity quantification of F12/F12a and plasminogen/plasmin (N = 6–12 microspheres, 2-experiments). Scale bar: 200 μm. **(D)** Assessment of plasmin activity on the microbeads after 4 h and 24 h (N = 3 donors). **(E)** The ratio of Δplasmin activity (plasmin activity T4 – T0)/ΔPTF1.2 (PTF1.2 T4 – T0) in 4 h.

Coagulation activation (Fig. 3A) was assessed by measuring the release of prothrombin fragment 1.2 (PTF1.2), which is generated upon the conversion of prothrombin (F2) to thrombin (F2a). All F/A microbeads induced significant amounts of PTF1.2 compared to SA/A, A and AP, irrespective of the fraction of fucoidan in F/A microbeads (Fig. 3B). Factor 12 (F12) is a crucial initiator of the intrinsic coagulation pathway and can be converted to active F12a by negatively charged surfaces [40]. Enrichment of F12/F12a on F/A microbeads was shown by CLSM imaging and corresponding intensity quantification (Fig. 3C). Likewise, plasminogen/plasmin was highly enriched on the surfaces of both F/A and SA/A microbeads. Importantly, the polyclonal antibodies against F12/F12a and plasminogen/plasmin do not distinguish between the native and active forms of these proteins. Therefore, a functional assay was used to monitor their surface-activated forms (plasmin and F12a as illustrated in Fig. 3A) by quantifying plasmin activity on the material surfaces. This demonstrated that both F/A and SA/A microbeads induced higher plasmin activity than A microbeads (Fig. 3D), with plasmin activities peaking between 4 and 24 h. Microbeads with lower fucoidan concentrations exhibited elevated plasmin activity compared to F/A 40/60 (Fig. 3D). The SA/A microbeads were as effective in activating plasmin as the most responsive F/A microbeads (Fig. 3D), despite binding less F12/F12a and plasmin/plasminogen on their surfaces (Fig. 3C).

The results from the functional assays may reflect the material's potential to induce fibrin formation and resolution, processes that are relevant to fibrosis. Thus, the ratio between the time-dependent change in plasmin activity and prothrombin fragment 1.2 (Eq. (1)), could serve as a predictor of the material's fibrosis-resistant properties. At 4 h, the *Δ*_*PA*_ T4*/Δ*_*PTF1.2*_ T4 ratio was elevated on the SA/A microbeads compared to F/A and A microbeads, indicating a higher fibrosis-resistant tendency of SA/A microbeads (Fig. 3E).

### 3.4 Fucoidan alginate microbeads show different cytokine signatures compared to sulfated alginate microbeads

**Fig. 4:**
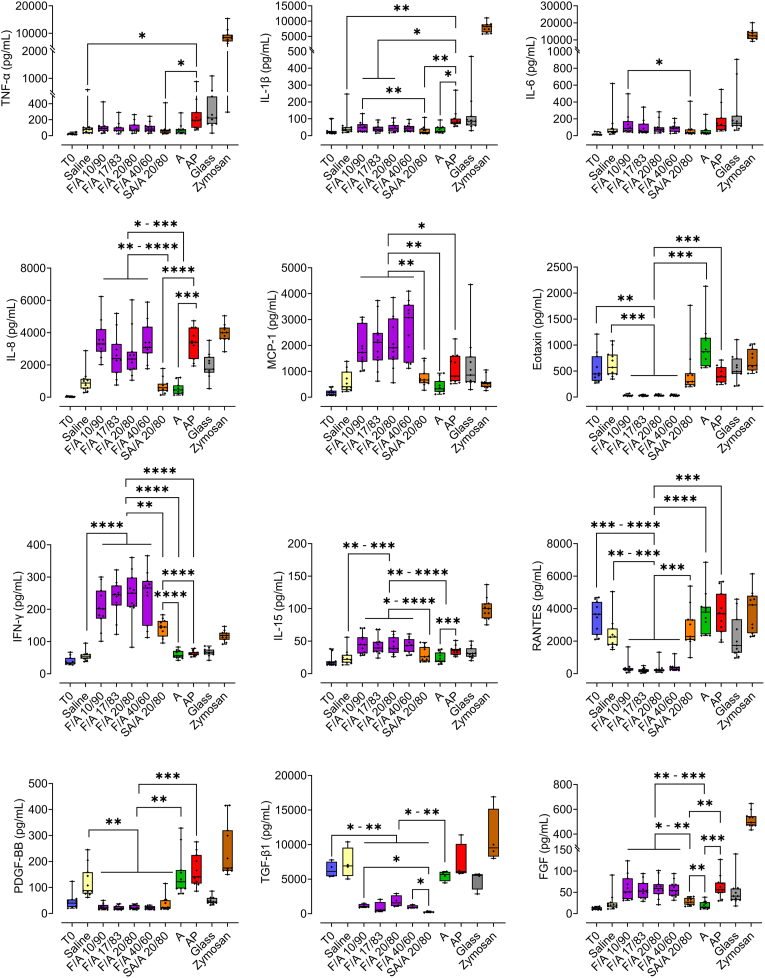
The potential of fucoidan alginate microbeads to induce cytokines, chemokines, and growth factors. Induction of cytokines after 4 h incubation of various microspheres and controls in whole blood anti-coagulated with hirudin (N = 11 donors). Values are given as mean with 95 % CI and significance as p ≤ 0.05 (∗), p ≤ 0.01 (∗∗), p ≤ 0.001 (∗∗∗), and p ≤ 0.0001 (∗∗∗∗) of fucoidan alginate microbeads (F/A) of various compositions as compared to either T0, Saline, SA/A, A and AP. T0 represents the experimental baseline. Data were analyzed by one-way ANOVA and Tukey HSD tests against relevant conditions.

The plasma concentrations of selected cytokines after 4 h of incubation in hirudin-anticoagulated human whole blood are shown in Fig. 4. The levels of pro-inflammatory cytokines TNF-α, IL-1β and IL-6 induced by F/A microbeads at 4 h were not significantly different from of the saline control or SA/A microbeads (Fig. 4). An exception was F/A microbeads containing the lowest fucoidan content (F/A 10/90), which elicited significantly higher levels of IL-1β (p ≤ 0.01), and IL-6 (p ≤ 0.05) compared to SA/A 20/80. IL-8, a key chemokine secreted by monocytes/macrophages and typically associated with pro-inflammatory responses, was induced at significantly higher levels by all F/A compositions compared to SA/A and A, consistent with the trend observed in TCC induction.

The monocyte chemoattractant protein-1 (MCP-1/CCL2), a pivotal cytokine involved in immune cell recruitment to inflammation sites, was significantly elevated (p-value between 0.05 and 0.01) by F/A compared to all other microbeads (Fig. 4 second row). Similarly, F/A induced higher levels of IFN-γ (p-value between 0.01 and 0.0001) than all other microbeads. Although lower than the F/A microbeads, SA/A also elicited significantly (p ≤ 0.0001) more IFN-γ than both A and AP (Fig. 4, third row). IL-15, a cytokine involved in the recruitment, differentiation and activation of immune cells, mainly T cells, was significantly (p-value between 0.05 and 0.0001) elevated by F/A compared to SA/A and A (Fig. 4).

Notably, some growth factors and chemokines were measured in significantly lower levels by F/A compared to the baseline (T0) or saline controls. This was observed for RANTES, Eotaxin (CCL11), PDGF-BB TGF-β1, TGF-β2 TGF-β3 and IL-7 (Fig. 4 and Supplementary Fig. S3). A potential explanation for this finding is that these factors bind to the F/A microbeads, thereby evading quantification.

Conversely, all F/A microbeads elicited significantly elevated levels of FGF (Fig. 4), G-CSF, GM-CSF and VEGF (p-value between 0.05 and 0.001) (Supplementary Fig. S3) compared to SA/A and A. All the microbeads mediated significantly (p-value between 0.05 and 0.0001) increased levels of the chemokines MIP-1α (CCL3) and MIP-1β (CCL4) compared to baseline, but no significant differences were observed between the different microbead types (Supplementary Fig. S3). AP elicited higher levels of inflammatory cytokines compared to the other microbeads, in agreement with previous findings [29,41].

Additionally, AP microbeads mediated stronger induction of growth factors compared to the other microbeads, while SA/A and A displayed lower cytokine induction (Supplementary Fig. S3). This trend was also observed for cytokines associated with T cell activation and differentiation, including IL-2, IL-4, IL-5, IL-9 and IL-17 (Supplementary Fig. S3). These results suggest that microbeads containing sulfated polysaccharides may have a generally lower potential to induce these cytokines, at least within a 4 h time frame.

### 3.5 Fucoidan alginate microbeads are more fibrotic than sulfated alginate microbeads

**Fig. 5:**
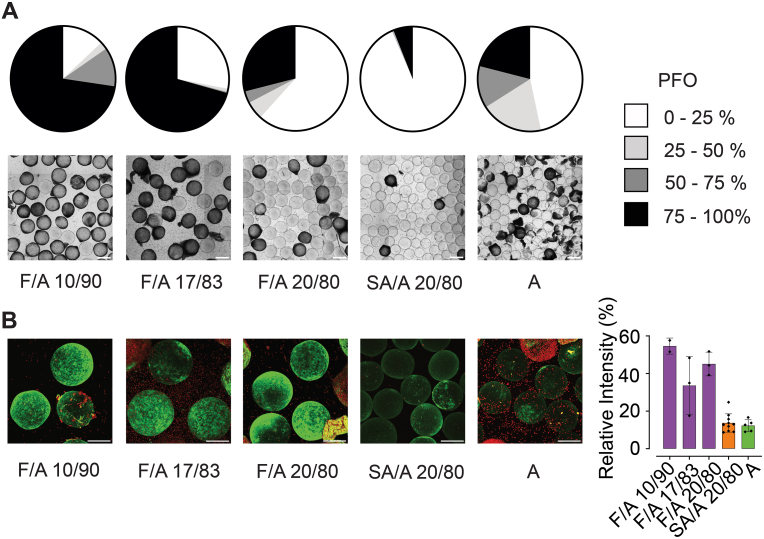
Pericapsular overgrowth (PFO) after 14 days of intraperitoneal implantation in C57Bl/*6*JRj mice. Alginate microbeads of fucoidan and alginate at various ratios (F/A 10/90, 17/83, 20/80) compared with sulfated alginate (SA/A 20/80) and alginate microbeads (A) explanted from mice (n = 2 – 3). **(A)** Pie charts with the average coverage of PFO for microspheres (n = 100) and representative brightfield images. Scale bar: 500 μm. **(B)** Deposition of fibrin(ogen) on microspheres with no PFO with Z-stack images of microbeads (left) and corresponding quantification of relative fluorescence intensity of fibrinogen (right). FITC-conjugated anti-fibrin(ogen) is displayed as green, and cell nuclei stained using DRAQ5 are displayed as red. Scale bar: 200 μm. (For interpretation of the references to color in this figure legend, the reader is referred to the Web version of this article.)

In this study, F/A with varying fucoidan concentrations were compared to A and SA/A 20/80 microbeads, which have previously been shown to mediate moderate and low levels of PFO, respectively [16,42]. The extent of PFO on F/A microbeads varied with fucoidan-to-alginate ratio (Fig. 5A). In brief, 13 % of F/A 10/90 and 28 % of F/A 17/83 microbeads displayed no or minimal (0–25 %) PFO, while 62 % of F/A 20/80 displayed no or minimal PFO. In comparison, 47 % of A and 93 % of SA/A microbeads displayed no or minimal PFO [16,42]. Fibrin(ogen) deposition was measured by CLSM and fluorescently labelled anti-fibrinogen antibodies (Fig. 5B). Overall, increased deposition of fibrinogen was found on F/A compared to A and SA/A microbeads.

### 3.6 Explanted fucoidan alginate and sulfated alginate microbeads show distinct patterns of protein adsorption revealed by LC-MS/MS

To elucidate protein patterns on explanted microspheres (after 24 h of implantation), we performed comprehensive protein profiling using high-resolution LC-MS/MS. Label-free quantification (LFQ) revealed a total of 3798 protein groups, with their relative abundance expressed as LFQ intensity values. To ensure robust identification of proteins, we applied an MS1 intensity threshold of 17.0 and required a minimum count of two peptides per protein. Given that activation of the complement and coagulation systems can contribute to PFO on microsphere surfaces [43], we focused on examining proteins involved in these pathways. Each protein detected on the SA/A 20/80 and F/A 20/80 microbeads was quantified relative to the AP microbeads, which are known to rapidly induce PFO and thus serve as a positive control [16].

**Fig. 6:**
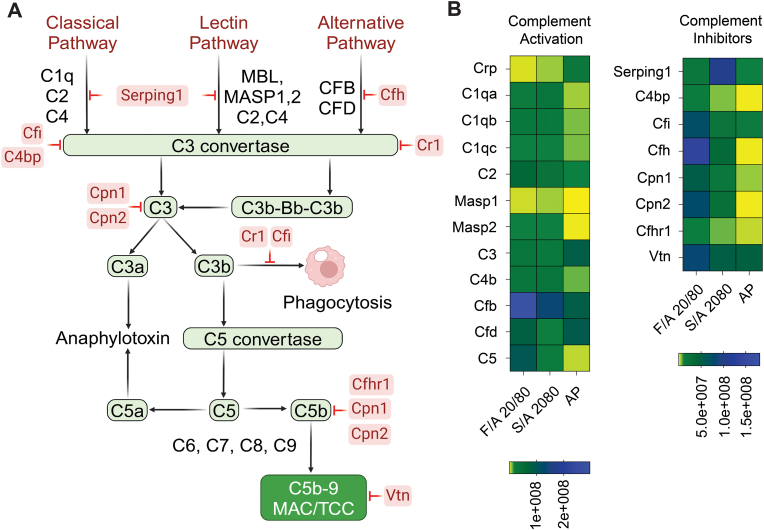
Complement activators and inhibitors on fucoidan alginate and sulfated alginate microspheres after 1-day intraperitoneal implantation in C57Bl/6JRj mice. **(A)** Schematic overview of the complement cascade with activators (black) and inhibitors (red) of mice, Created with BioRender.com. **(B)** Heatmap showing identified complement system activator and inhibitor proteins adsorption profiles on the microspheres containing fucoidan (F/A 20/80), sulfated alginate (SA/A 20/80) and alginate-poly-lysine (AP). Protein abundances are colored based on label-free quantification (LFQ) intensity values, indicated by color scale bars (N = 4 technical replicates, 1 mouse). (For interpretation of the references to color in this figure legend, the reader is referred to the Web version of this article.)

Fig. 6 provides an overview of complement activators and inhibitors (Fig. 6A) and depicts the protein abundance of selected activators (Fig. 6B) and inhibitors (Fig. 6C) as heat maps based on LFQ intensity values. Notably, the C1q complex subunits C1qa, b, and c, which initiate the classical pathway, were significantly enriched on F/A 20/80 (13-, 12-, and 4-fold, respectively) and SA/A 20/80 (20-, 10-, and 4-fold, respectively) compared to AP microbeads (Fig. 6B). The lectin pathway proteins, Masp1 and Masp2, were also enriched on SA/A 20/80 (58- and 47-fold, respectively) and F/A 20/80 microbeads (25- and 49-fold, respectively) (Fig. 6B). Additionally, C2 protein was enriched on the sulfated and fucoidan microbeads (6 and 11-fold, respectively) (Fig. 6B). These proteins are inhibited by the plasma protease C1 inhibitor, (Serping1) which was markedly more enriched on SA/A 20/80 (25-fold) than on F/A 20/80 (4-fold) (Fig. 6B). This suggests that SA/A microbeads have a greater potential to inhibit classical and lectin-pathway activation.

In the alternative pathway, complement factor b (Cfb) was identified on all microbeads, with the highest enrichment on F/A 20/80 (3-fold) and SA/A 20/80 (1.6-fold) compared to AP. Conversely, complement factor D (Cfd) was most enriched on AP (10-fold compared to SA/A 20/80 and 1.1-fold compared to F/A 20/80). C1q, Masp1/2 and Cfb/Cfd potentially contribute to the activation of complement component, C3, which leads to downstream activation of C5, culminating in the formation of TCC. AP exhibited the highest enrichment of C3 (4-fold compared to SA/A 20/80 and 3-fold to F/A 20/80). Key inhibitors of C3 activity, such as C4bp, Cfi, and Cfh, displayed strong enrichment on microbeads containing sulfated polymers: SA/A 20/80 (234-fold) and F/A 20/80 (901-fold) for Cfh, while Cfi and C4bp showed moderate enrichment on SA/A 20/80 (2- and 15.5-fold, respectively) and F/A 20/80 (6 and 80-fold, respectively). Other C3 inhibitors enriched on the microbeads were Cpn1 (12-fold on SA/A 20/80 and 37-fold on F/A 20/80) and Cpn2 (200-fold on SA/A 20/80 and 602-fold on F/A 20/80). Cpn1/2 degrades anaphylatoxins (C3a and C5a) to their des Arg forms. The C5 protein, a key component of the TCC, was significantly enriched on SA/A 20/80 and F/A (15- and 102-fold, respectively). Inhibitors crucial for TCC assembly, such as complement factor H-related protein 1 (Cfhr1) and vitronectin (Vtn), were enriched on SA/A 20/80 and F/A 20/80, with Cfhr1 showing a 2.5 and 10-fold increase, respectively, and Vtn showing a 1.1 and 1.7-fold increase, respectively.

**Fig. 7:**
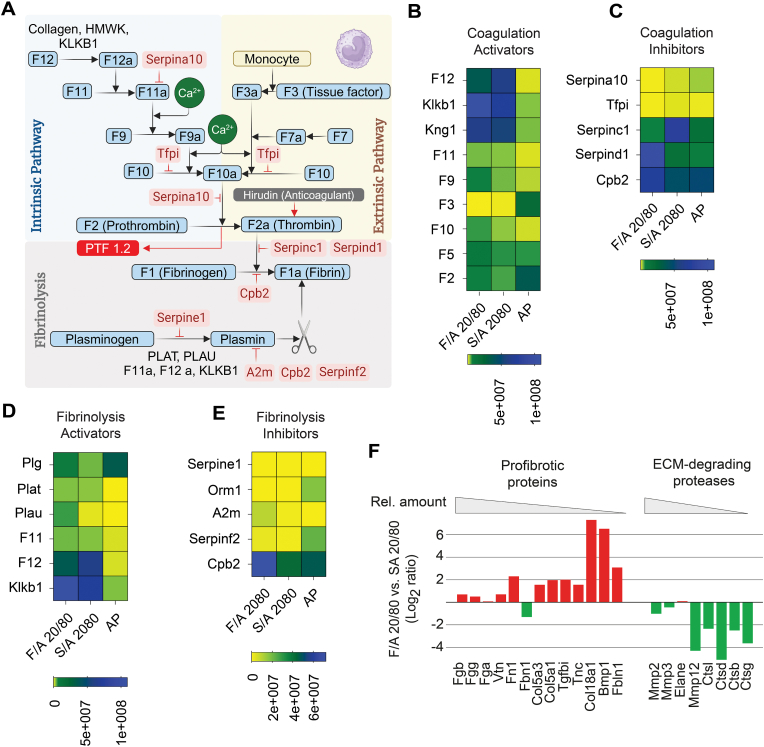
Activators and inhibitors of coagulation, fibrinolysis and profibrotic/ECM-degrading protein profiles on fucoidan alginate and sulfated alginate microspheres after 1-day intraperitoneal implantation in C57Bl/6JRj mice. **(A)** Schematic overview of the coagulation system with the main activators (black), inhibitors (red), and fibrinolytic pathway in mice, Created with BioRender.com. **(B)** Heatmap showing identified activator and **(C)** inhibitor proteins of the coagulation pathway and fibrinolysis pathway activators **(D)** and inhibitors **(E)** on the microspheres. The enrichment pattern of profibrotic proteins and ECM-degrading non-plasmin proteases are shown as a comparison between SA/A 20/80 and F/A 20/80 microbeads **(F)**. Protein abundances are colored based on label-free quantification (LFQ) intensity values, indicated by a color scale bar. (N = 4 technical replicates, 1 mouse). (For interpretation of the references to color in this figure legend, the reader is referred to the Web version of this article.)

Fig. 7A summarizes the proteins involved in the coagulation cascade and fibrinolysis, with their LFQ intensities shown as heat maps (Fig. 7B–E). Proteins in the intrinsic pathway [F12, F11, kallikrein (Klkb1), and high-molecular-weight kininogen (Kng1)] were detected on all microspheres, and enriched on SA/A 20/80 and F/A 20/80 (F11: 3.3 and 3.8-fold, F12: 149 and 88-fold, Klkb1: 56 and 80-fold, Kng1: 169 and 201-fold) compared to AP. Factor 9 (F9), activated by F11, exhibited enrichment on SA/A 20/80 (1.9-fold) and F/A 20/80 (5.2-fold). Conversely, the extrinsic pathway factor 3 (F3; Tissue factor) was strongly enriched on AP relative to the other microbeads (121.1-fold compared to SA/A 20/80 and 242.7-fold compared to F/A 20/80). F3 binds to F7 and activates F10, which is common to both pathways. F10 was enriched on SA/A 20/80 (2.3-fold) and F/A 20/80 (4.1-fold) compared to AP. Subsequently, thrombin (F2) activates and converts fibrinogen (Fga) to fibrin. F2 was enriched on AP (18.1-fold compared to SA/A 20/80 and 9-fold compared to F/A 20/80) (Fig. 7B). F5, a potential activator of F2, was adsorbed to a similar extent to the different beads. Fibrinogen subunits were enriched on SA/A 20/80 and to an even greater extent on F/A 20/80 (Fga: 6.9 and 7.3-fold, Fgb: 16.3 and 26.3-fold, Fgg: 15 and 22-fold).

Among coagulation system inhibitors, Serpina10, an inhibitor of F10, was enriched on AP relative to SA/A (2.1-fold) and F/A (13.1-fold) microbeads. Serpinc1 and d1, both inhibitors of F2a, showed selective enrichment on SA/A 20/80 (5.8-fold) and F/A 20/80 (24.8-fold) compared to AP. Cpb2, which is a plasmin-activated inhibitor of fibrinolysis [44,45], was present on all microspheres, particularly on F/A 20/80 (1.5-fold compared to AP).

Fibrinolysis, the process of degrading coagulation products, is mediated by the protease enzyme, plasmin (Plg), which is derived from native plasminogen. Our analysis revealed the enrichment of fibrinolysis activator proteins on SA/A (Plat: 593-fold, Plau: 40.2-fold, F11: 3.2-fold, F12: 149.3-fold, and Klkb1: 56.1) and F/A 20/80 (Plat: 661-fold, Plau: 365-fold, F11: 3.7-fold, F12: 87.9-fold, and Klkb1: 79.8-fold) compared to AP. Conversely, the plasmin inhibitors Orm1 and Serpinf2 were markedly enriched on AP compared to both SA/A (1296.7- and 24.1-fold, respectively) and F/A (13770.7- and 8.2-fold, respectively).

Fibrotic tissues contain various extracellular matrix (ECM) proteins, including collagens, fibronectin, vitronectin and fibrillin [46], which influence the biophysical properties and cell-matrix interactions. Their levels are regulated by ECM-degrading proteases, such as cathepsins, metalloproteinases and neutrophil elastase. Fig. 7F shows the log_2_-fold ratios of the most abundant profibrotic proteins as well as non-plasmin ECM-degrading factors on explanted F/A 20/80 versus SA/A 20/80 microbeads. While the various factors dictating fibrin deposition and resolution showed a mixed pattern of adhesion to the microbeads, the ECM proteins demonstrated a consistently increased adhesion to the F/A 20/80 microbeads. An exception to this was fibrillin (Fbn1), which was selectively enriched on SA/A 20/80. However, the accumulation of Fbn1 may not be directly associated with fibrosis, as an experimental mouse model of chronic kidney disease demonstrated that depletion of Fbn1 improved renal fibrotic lesions [47]. Consistent with the selective accumulation of fibrotic ECM proteins, F/A 20/80 microbeads exhibited an overall reduced binding of ECM-degrading proteases. We thus hypothesize that the accumulation of profibrotic ECM proteins is a major determinant of PFO on fucoidan-based microbeads *in vivo*.

## 4 Discussion

In this study, we investigated the host responses to alginate microbeads containing fucoidan from *Laminaria hyperborea*. The biological properties of fucoidan-alginate microbeads were compared to those of microbeads containing sulfated alginate, produced through chemical modification. Although both polysaccharides contain sulfate groups, they differ significantly in their structure with respect to monomer composition, the arrangement of sulfate groups and several other structural features [4,24]. Fucoidan has a branched structure with sulfate groups attached at various positions along the polysaccharide chain in a non-repeating fashion [4]. In contrast, sulfated alginate has a simpler, well-established linear structure, with sulfate groups distributed along the linear polysaccharide chain. Our results demonstrate that the fucoidan-alginate microbeads exhibited more pro-inflammatory and pro-fibrotic activities when compared to the sulfated alginate microbeads. Furthermore, distinct protein adsorption patterns were found on the surfaces of the microbeads, which could potentially explain differences in the host responses.

Microbeads with fucoidan induced prominent complement activation, coagulation, and PFO, in contrast to microbeads containing sulfated alginate. The complement activation contrasts our previous findings for the same fucoidan material in solution, where no activation was observed across different concentrations of fucoidan [4]. The fucoidan characterized by Kopplin et al., [4] and used in the present study primarily consists of fucose residues with a flexible 1 → 3 backbone. Compared to sulfated alginate (D.S. = 0.83, Mw = 162 kDa), this fucoidan exhibits approximately twice the degree of sulfation (D.S. = 1.7) and a substantially higher molecular weight (Mw = 469 kDa) [4]. To provide comparable polymer concentrations and sulfate group levels in the microbeads, varying ratios of fucoidan and sulfated alginate were incorporated. While some of the investigated host responses (coagulation, plasmin activity, and PFO) appeared to be concentration-dependent for fucoidan in alginate microbeads, the main differences were observed between the fucoidan-containing samples and the sulfated alginate-containing samples, regardless of fucoidan concentration. These differences in host responses between sulfated alginate and fucoidan in alginate hydrogels are challenging to attribute exclusively to polymer structure or concentration effects, as both this fucoidan and sulfated alginate in solution have been shown to produce similar complement and coagulation responses [4]. In contrast to fucoidan, sulfated alginate has gelling capabilities, albeit weaker than those of native alginate [43]. Hence, differences in the binding properties of the sulfated polysaccharides and their presentation at the hydrogel surface may contribute to the biological differences observed in this study. Physical characteristics such as size and shape can also exert a significant impact on the bioactivities such as foreign body responses [48]. Similarly sized and shaped microspheres were employed in this study to rule out the variations related to these physical parameters.

Fucoidan-alginate microbeads activated complement and coagulation in the whole blood assay. Interestingly, proteomic and CLSM data revealed significant enrichment of both complement and coagulation inhibitors. Together, these results suggest a dynamic interaction of acute phase factors on the microbead surfaces, involving both activating and counterbalancing proteins. Previous findings have shown that the activation potency of fucoidan in solution for both complement and coagulation systems varies significantly with its molecular weight, degree of sulfation, and polymer concentration; higher sulfation and high molecular weight and higher concentrations resulted in increased inhibitory effects [4]. This aligns with current functional trends for coagulation and binding of both complement by CLSM data (activator C1q and inhibitors C1-INH, FH) and coagulation (F12), showing a dependency on the proportions of fucoidan to alginate. The effect of concentration was less evident for complement where all concentrations of fucoidan resulted in robust complement activation. In contrast, the amount of sulfated alginate in the alginate microbeads did not result in complement activation for the concentrations studied. The data herein may thus suggest that the functional outcomes of sulfated alginate may be less dependent on the concentration in the microbeads.

Proteomic analysis following early-stage intraperitoneal implantation in mice revealed further the complexity of activating and counteracting inhibitory factors on both fucoidan and sulfated alginate microbeads. Proteins from all three complement pathways were identified, including classical pathway components (C1q a, b, c, C2), lectin pathway factors (Masp1, 2), and alternative pathway proteins (Cfb, Cfd). The key inhibitor of the classical and lectin pathways, C1-INH (Serping1), was significantly more enriched (∼6-fold) on sulfated alginate than on fucoidan-containing microbeads. Conversely, C3 convertase inhibitors (C4bp, Cfi, Cfh) were more abundant on fucoidan-alginate microbeads compared to sulfated alginate microbeads, with both showing greater enrichment than AP. These inhibitors likely reduce C3-convertase deposition and activation on fucoidan- and sulfated alginate microbeads more effectively than on AP. The current data from mice aligns with previous proteomic data from human lepirudin plasma, which demonstrated that sulfated alginate microbeads exhibit a high affinity for human complement inhibitors [19]. We currently reaffirm the main findings by CLSM, showing high enrichment of the inhibitors C1-INH and FH, but also the classical pathway activator C1q. A discrepancy between CLSM and proteomic findings showed higher C1-INH on fucoidan alginate microbeads, possibly due to different experimental conditions. Despite these discrepancies, the data overall highlights a balance between complement activators and inhibitors on the microbeads, influencing complement pathway regulation. Notably, the inhibition of C3 did not reduce the enrichment of FH, suggesting that FH binds directly to the sulfated polysaccharides.

Coagulation can be activated through F12/kallikrein (intrinsic pathway) or indirectly via tissue factor (TF) pathway, involving monocytes and complement activation [33]. Prominent F12/F12a staining, as visualized on fucoidan alginate microbeads by CLSM, suggests that the intrinsic pathway could be the primary driver of coagulation. Lower F12/F12a levels on sulfated alginate microbeads could explain their reduced coagulation potential. Previous proteomic analyses using a lepirudin-based human plasma model demonstrated that sulfated alginate microbeads are enriched with coagulation inhibitors [19]. The current proteomic data from mice revealed that both fucoidan- and sulfated alginate microbeads enrich intrinsic pathway activators (F12, kallikrein B, Klkb1, HMW-kininogen; Kng1, F11, and F9) and late-stage inhibitors (Serpinc1, Serpind1, Cpb2) to varying extents. The differences in coagulation outcomes in the functional assay reflect the complex interplay between coagulation activators and inhibitors associated with these microbeads.

Both sulfated polysaccharide microbead surfaces were found to mediate plasmin activity, which is associated with fibrinolysis. Fibrinolysis balances fibrin formation during coagulation by degrading fibrin clots through plasmin activity. Fibrin formation might be a foundation triggering fibrosis [16,49]. Previously, we suggested that a surface that promotes plasmin activity could reduce fibrin formation [19]. In the current study, sulfated alginate microbeads more effectively mitigated fibrin/fibrinogen deposition and fibrosis, manifested as pericapsular fibrotic overgrowth (PFO), thereby reaffirming our previous findings [16]. While both fucoidan- and sulfated alginate microbeads promoted plasmin activity, the ratio of plasmin activity to coagulation activation was significantly higher by sulfated alginate microbeads. These differences corresponded with the fibrotic outcomes. Since these parameters respectively reflect fibrin formation (coagulation) and degradation (plasmin), we propose that this metric could serve as a valuable predictor for identifying materials with fibrosis-resistant properties.

The current proteomic data obtained after intraperitoneal implantation in mice provide further insights into the process of fibrinolysis. Fibrinolysis depends on the activation of plasminogen to plasmin, which can occur through F12, kallikrein-kinin (Klkb1, Kng1), and tissue plasminogen activator (tPA) [50,51], as illustrated in Fig. 7A. Notably, both fucoidan- and sulfated alginate-containing microbeads were enriched with these factors, potentially promoting plasmin activation. In contrast, the fucoidan-containing microbeads exhibited higher levels of plasmin inhibitors (Cpb2, A2m), suggesting reduced fibrinolytic activity on their surfaces. This observation aligns with the higher fibrotic depositions on these microbeads.

Notably, the proteomic findings further demonstrated that the sulfated alginate microbeads had a lower affinity for pro-fibrotic proteins and an increased binding of ECM-degrading proteases compared to fucoidan-containing microbeads. ECM-degrading proteases, including metalloproteinases (Mmp2, Mmp3, Mmp12) and cathepsins (Ctsl, Ctsd, Ctsb, Ctsg), were found in lower amounts on fucoidan-containing than sulfated alginate microbeads. Furthermore, the fucoidan-containing microbeads were enriched with fibrosis-promoting proteins (Fbg, Fgg, Fga, Vtn, Fn1, Col5a3, Col5a1, Tgfbi, Tnc, Col18a1, Bmp1, Fbln1). Thus, the balance between ECM-degrading and promoting proteins appears to achieve a distribution more favourable to low PFO on sulfated alginate microbeads. This further suggests that materials enriching ECM-degrading proteases could resist fibrosis. The observed concentration-dependent effects of fucoidan on fibrotic potency, as indicated by PFO findings and whole blood outcomes, suggest that the binding of profibrotic or ECM-degrading proteins may also vary with concentrations. However, the scope of proteomic analyses was limited to a single fucoidan-alginate microbead type (F/A 20/80), restricting our ability to reveal potential concentration-dependent binding on distinct microbead surfaces.

Sulfate groups in polysaccharides, commonly found in the extracellular matrix (ECM), play crucial biological roles through their anionic charge and electrostatic interactions with proteins [50,52]. Their diverse bioactivities include both pro- and anti-inflammatory responses [53]. For instance, sulfation of hyaluronan, a naturally unsulfated ECM polysaccharide, has been demonstrated to enhance its anti-inflammatory properties by inhibition of cytokines such as MCP-1, IL-6 and TNF-α [54]. This effect is correlated with the degree of sulfation, highlighting the role of sulfate groups in immunomodulation. Interactome analysis of heparan sulfate and heparin, sulfated polysaccharides present in the ECM, demonstrated the binding of a wide spectrum of proteins including complement proteins (C3a, C5a, CFH), chemokines, growth factors (FGF, PDGF, TGF) and apolipoproteins [55]. Similarly, we have recently shown the adsorption of heparin-binding proteins to alginate beads containing sulfated alginate [19]. Hence, these findings suggest similarities in protein-binding and biological activities among sulfated polysaccharides.

Current data indicate distinct cytokine patterns associated with the various materials, reflecting either cytokine binding and/or induction. The distinct cytokine patterns by fucoidan-alginate microbeads exhibited the most prominent reductions in cytokines PDGF-BB, TGF-β isoforms, RANTES, and Eotaxin, along with inductions of IL-8, MCP-1, IFN-γ and IL-15. The lower cytokine levels observed compared to baseline could be due to specific interactions between functional groups and cytokine molecules, as corroborated by TGF-β binding in the current proteomic study. TGF-β isoforms are known to bind to and be activated by sulfated polysaccharides such as heparin [56,57], and both fucoidan and sulfated alginate in solution have previously demonstrated characteristics akin to heparin [4,24]. TGF-β commonly plays a role in promoting fibrosis [58], and therapeutic strategies targeting TGF-β have shown efficacy in reducing fibrotic scar formation [59]. In our whole blood data, fucoidan-alginate and sulfated alginate microbeads elicited low TGF-β levels. However, in mice, more TGF-β was found adsorbed on the fucoidan-alginate microbeads compared to sulfated alginate microbeads. Hence, we cannot rule out the relevance of TGF-β binding in contributing to the differences in fibrotic outcome (PFO) observed in this study.

The elevated levels of IL-8 and MCP-1 may be linked to complement activation, as we previously demonstrated with poly-L-lysine coated alginate microbeads [41]. Previously, we have shown that interactions between complement C3 attached to the microbead surface and leukocyte CR3 receptor (CD11b/CD18) could lead to the induction of a broad range of cytokines, including IL-1β, TNF-α and IL-6 [29,41]. In the current study, we observed a low induction of these cytokines and reduced C3 deposition on fucoidan-alginate and sulfated alginate microbeads compared to poly-L-lysine coated alginate microbeads. This suggests that bound C3 on the fucoidan and sulfated alginate surfaces is likely in naïve form. One potential explanation for the elevated IL-8 and MCP-1 levels could be their induction by activated complement factors present in the fluid phase. This explanation is supported by previous findings that the terminal complement complex (TCC) can induce IL-8 and MCP-1 [60]. These distinct induction patterns between fucoidan- and sulfated alginate-containing microbeads may therefore be linked to the differences in complement activation. Additionally, IL-8 and MCP-1 are strong chemoattractants for neutrophils [61] and monocytes [62], respectively. IFN-γ, critical for innate and adaptive immunity, was elevated for both fucoidan and sulfated alginate microbeads, suggesting the involvement of NK/NKT cells in early immune responses. Similarly, IL-15, which activates NK and T cells, was higher with fucoidan-alginate microbeads, potentially indicating a greater capacity to activate NK cells. The relative increase of IFN-γ with fucoidan compared to sulfated alginate microbeads may be attributed to the upregulation of IL-15, a cytokine known to enhance IFN-γ production from activated NK and T cells [63,64]. The secreted IFN-γ may drive monocyte differentiation towards the proinflammatory, short-lived macrophage phenotype, M1 [65]. Since macrophages are the key drivers of fibrosis, including foreign body reactions [66], a phenotypic change into short-lived types might be advantageous in reducing these responses. However, this transition might not be fully elucidated within a short-term (4 h) human whole blood model, as the *in vivo* differentiation of monocytes into macrophages typically occurs over an extended timeframe (1–4 days) [66].

Recent studies highlight the role of lipid deposition in modulating foreign body responses to biomaterials [51]. Phospholipid deposition on transplanted materials upregulates anti-inflammatory genes in macrophages, whereas fatty acids induce pro-inflammatory gene expression [51]. Our proteomic analysis revealed a significant enrichment of apolipoproteins (Apoa1, Apoa2, Apob, Apoc2, ApoE and ApoH) on fucoidan-alginate and sulfated alginate microbeads compared to alginate-poly-L-lysine capsules (Supplementary Fig. S4). Apolipoproteins such as Apoa1, ApoE and ApoH, which showed significant enrichment on fucoidan-alginate microbeads, possess both inflammatory and anti-inflammatory properties [[67], [68], [69]]. Apolipoproteins are known to transport proteins, including inhibitors of complement and coagulation pathways [70], potentially influencing the immunomodulatory characteristics of the fucoidan alginate microbeads. However, the specific roles of these apolipoproteins in immune responses need further investigation.

The *ex vivo* human whole blood model is recognized for its physiological relevance but lacks the ability to depict fibrin formation due to thrombin inhibition, which could be an important starting point of fibrosis [16]. This study highlights the significance of the ratio between plasmin activity and coagulation as a predictor of fibrosis, aligned with the outcome in the C56BL/6JRj model. Despite limitations in predicting human physiology completely, this mouse model demonstrates improved translatability to larger non-human primates [71,72]. The current proteomic approach does not distinguish between absorbed and activated proteins but effectively details protein profiles. In current study it also showed enrichment profiles aligned with fibrosis outcomes.

Clinical transplantations of human pancreatic islets and clinical-grade human hepatocytes within alginate microbeads have demonstrated safety but still promoted fibrosis over the long term [[14], [15]]. These microbeads, composed of native alginate have shown a low-inflammatory profile in human whole blood studies but promoted fibrosis in mice and non-human primate models [16,29,32,72]. Although sulfated alginate microbeads demonstrate a lower inflammatory and fibrotic signature compared to native alginate microbeads, they have not yet been validated in non-human primates or clinical trials. To our knowledge, this is the first study addressing inflammatory and fibrotic responses to fucoidan-alginate microbeads. While the proinflammatory and profibrotic effects of fucoidan in the alginate hydrogels may limit its use in cell therapy, the distinct host responses detailed in our study could inform its application in other areas. Overall, the distinct patterns observed in the current study suggest that sulfated polysaccharides with varying properties could be selectively applied in tissue engineering and wound healing applications. This highlights the need to investigate each material for its intended use.

## 5 Conclusions

Here, we demonstrate that composite hydrogels of fucoidan and alginate activate coagulation, complement, and plasmin activity, along with distinct cytokine profiles in an *ex vivo* human whole blood model. The functional outcomes of fucoidan-alginate hydrogels differ from those of sulfated alginate, particularly in complement and coagulation activation, distinct cytokine induction profiles, and fibrosis potential. Both materials exhibit plasmin activity, which counteracts overall fibrin formation. However, fucoidan mediates stronger coagulation compared to sulfated alginate. Thus, the ratio between plasmin activity and coagulation apparently represents the balance of fibrin degradation versus formation, serving as a predictor of fibrosis.

Protein profiling by LC-MS/MS and CLSM revealed that these materials enrich a complex array of activators and inhibitors related to complement, coagulation, fibrinolysis, and extracellular matrix proteins. Fucoidan alginate hydrogels exhibited an overall strong ability to bind complement and coagulation inhibitors. The enhanced binding of ECM-degrading proteases and the reduced presence of profibrotic proteins on sulfated alginate microbeads, underline that the sulfated alginate hydrogels are more effective in resisting fibrosis compared to fucoidan alginate hydrogels.

The distinct patterns of growth factor binding and cytokine induction underscore the unique biological activities of fucoidan alginate hydrogels. While sulfated alginate is promising for long-term cell encapsulation, fucoidan alginate hydrogels may have potential applications in tissue engineering and wound healing due to their distinct bioactivity.

Overall, this study highlights the divergent functional profiles of sulfated polysaccharides in alginate hydrogel microbeads, underscoring that materials with similar functional groups but differing structures can elicit variable biological responses, depending on the balance between activating and inhibiting factors.

## CRediT authorship contribution statement

**Kalaiyarasi Vasuthas:** Writing – review & editing, Writing – original draft, Visualization, Methodology, Investigation, Formal analysis, Data curation. **Joachim Sebastian Kjesbu:** Writing – review & editing, Writing – original draft, Visualization, Methodology, Investigation, Formal analysis, Data curation. **Alessandro Brambilla:** Writing – original draft, Visualization, Software, Methodology, Investigation, Formal analysis, Data curation. **Maya Levitan:** Writing – original draft, Formal analysis. **Abba Elizabeth Coron:** Writing – review & editing, Project administration, Methodology, Investigation. **Davi M. Fonseca:** Software, Methodology. **Berit L. Strand:** Writing – review & editing, Project administration, Investigation, Funding acquisition, Conceptualization. **Geir Slupphaug:** Writing – review & editing, Resources, Investigation, Funding acquisition, Data curation, Conceptualization. **Anne Mari A. Rokstad:** Writing – review & editing, Supervision, Resources, Project administration, Methodology, Investigation, Funding acquisition, Data curation, Conceptualization.

## Funding

This work was supported by the 10.13039/100009123Norwegian University of Science and Technology (10.13039/100009123NTNU) Health project, The Liaison Committee for Education, Research, and Innovation in Central Norway (Regional Health Authority) under grant 30398, CEMIR NFR grant 223255, and the Chicago Diabetes Project (www.chicagodiabetesproject.org), and Stiftelsen Biopolymer. PROMEC is funded by the Faculty of Medicine at NTNU and 10.13039/501100004590Central Norway Regional Health Authority. PROMEC is a member of the National Network of Advanced Proteomics Infrastructure (NAPI), which is funded by the RCN INFRASTRUKTUR-program (295910). Data storage and handling were supported under the PRIDE and Norstore/Notur projects NN9036K/NS9036K, respectively.

## Declaration of competing interest

The authors declare that they have no known competing financial interests or personal relationships that could have appeared to influence the work reported in this paper.

## Data Availability

Data will be made available on request.
