# Supplementary material for: Fucoidan alginate and sulfated alginate microbeads induce distinct coagulation, inflammatory and fibrotic responses
Source: Mater Today Bio. 2025 Jan 10;31:101474. doi: 10.1016/j.mtbio.2025.101474 (PMC11783016; doi:10.1016/j.mtbio.2025.101474)
Supplement: Multimedia component 1 [file mmc1.docx]

# Supplementary Figure 1 (Fig. S1)


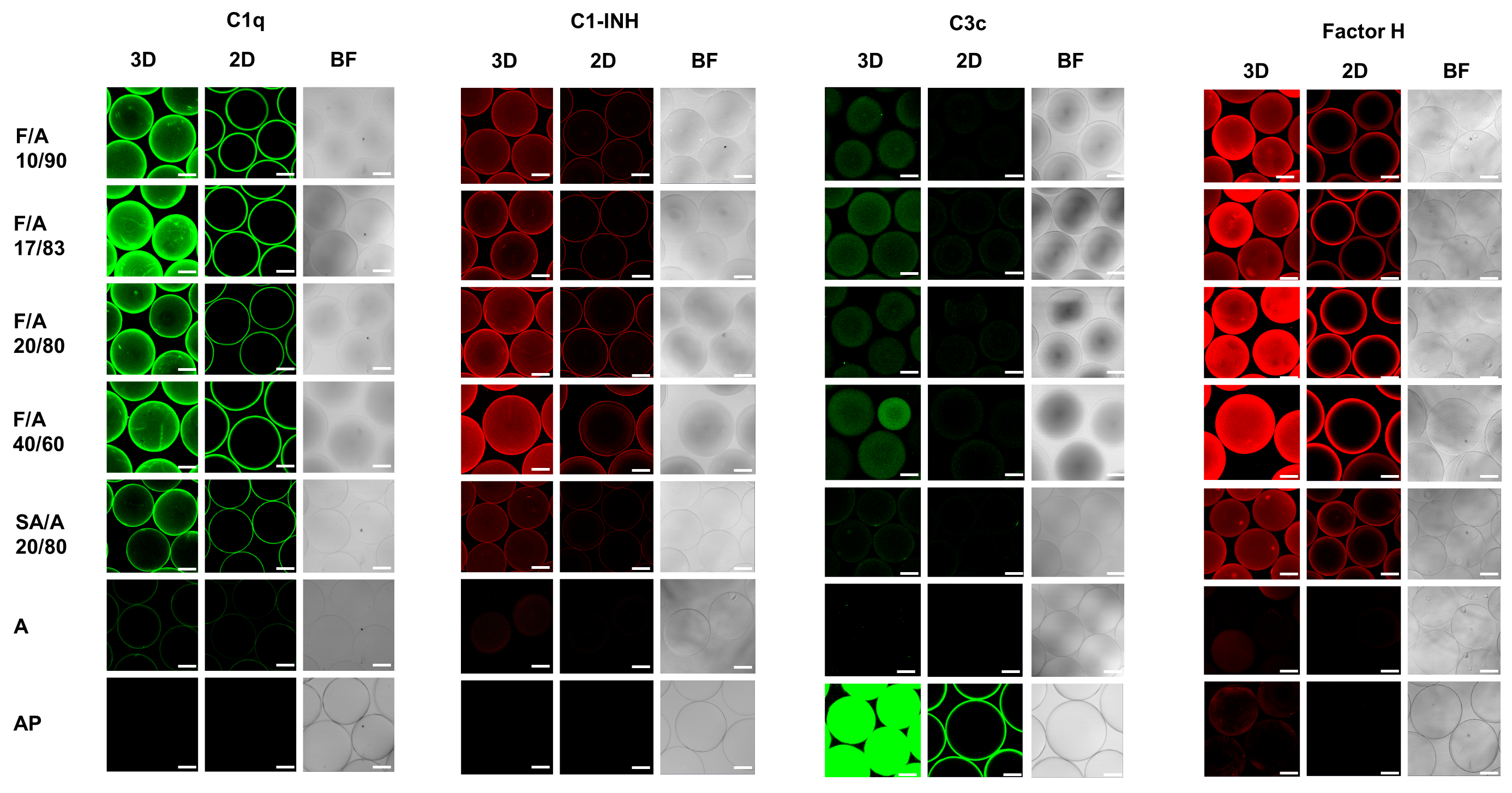


D

C

B

A

***Fig. S1****: Evaluation of complement and coagulation proteins and responses on/to microspheres. Detection of complement (C1q, C1 inhibitor, C3c, factor H) in human plasma by CLSM (A-D). CLSM images show antibody-stained microspheres after incubation (24 h) in hirudin-anticoagulated human plasma. Microspheres were FITC-stained (green) against C1q (A) and C3c (B), Alexa Fluor 647-stained (red) against C1inh (C), and CF633-stained (red) against FH (D) and non-specific antibody binding was assessed. Captured images include brightfield (BF), equatorial (2D)-sections and (3D)-projections of z-stacked images. Scale bar = 200 µm.*

# Supplementary Figure 2 (Fig. S2)

**D**

**A**

**B**


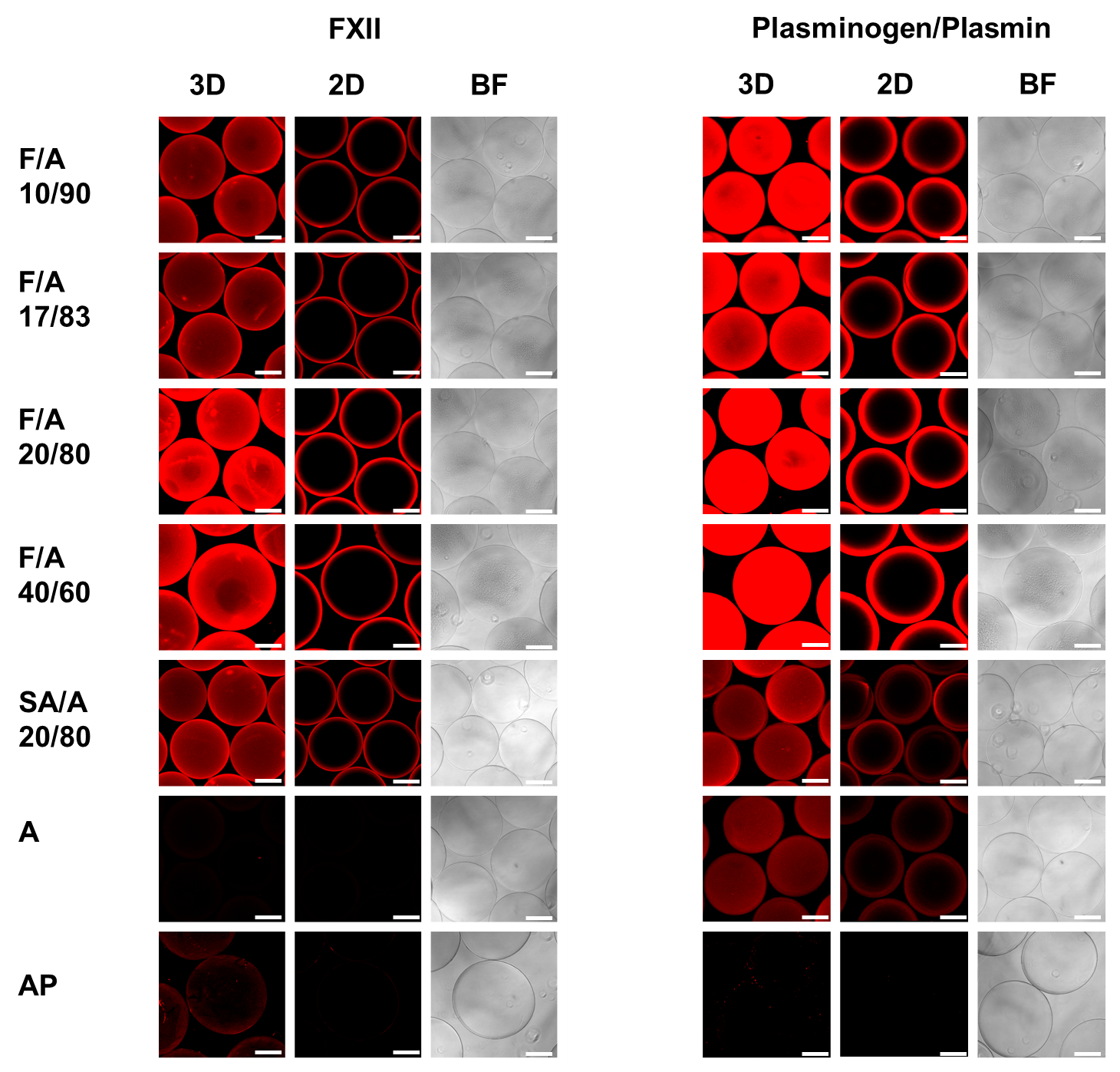

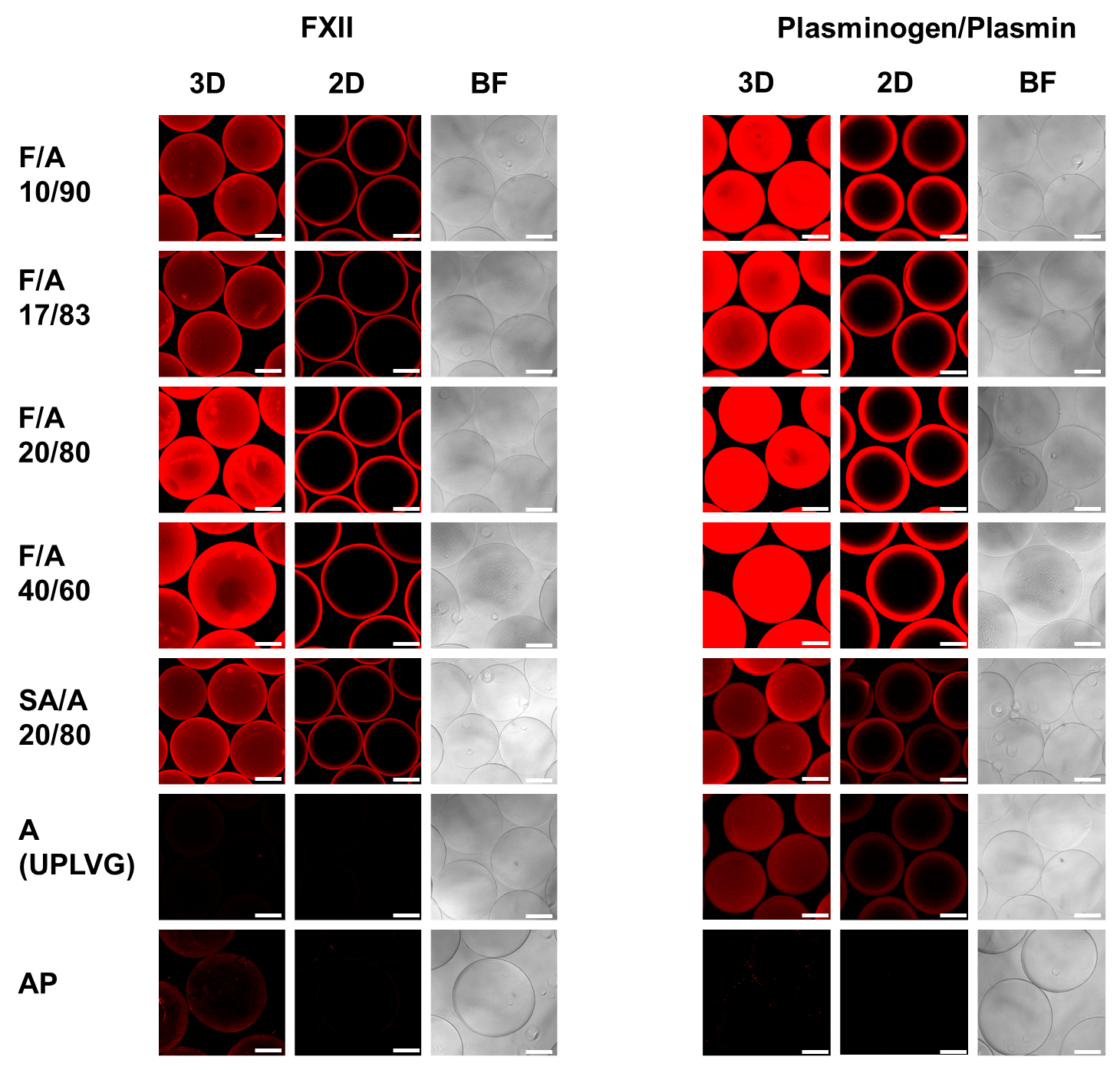


**C**


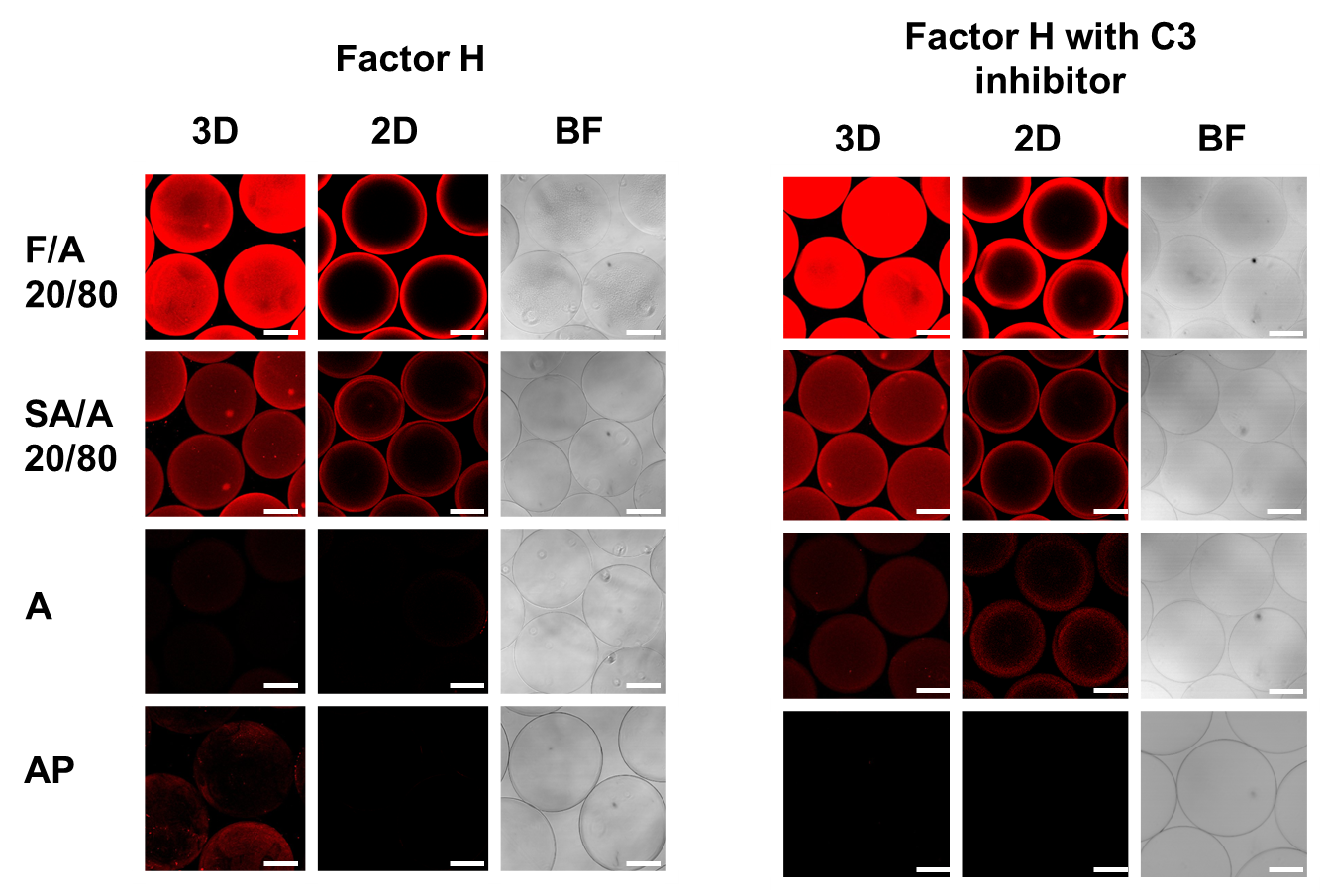

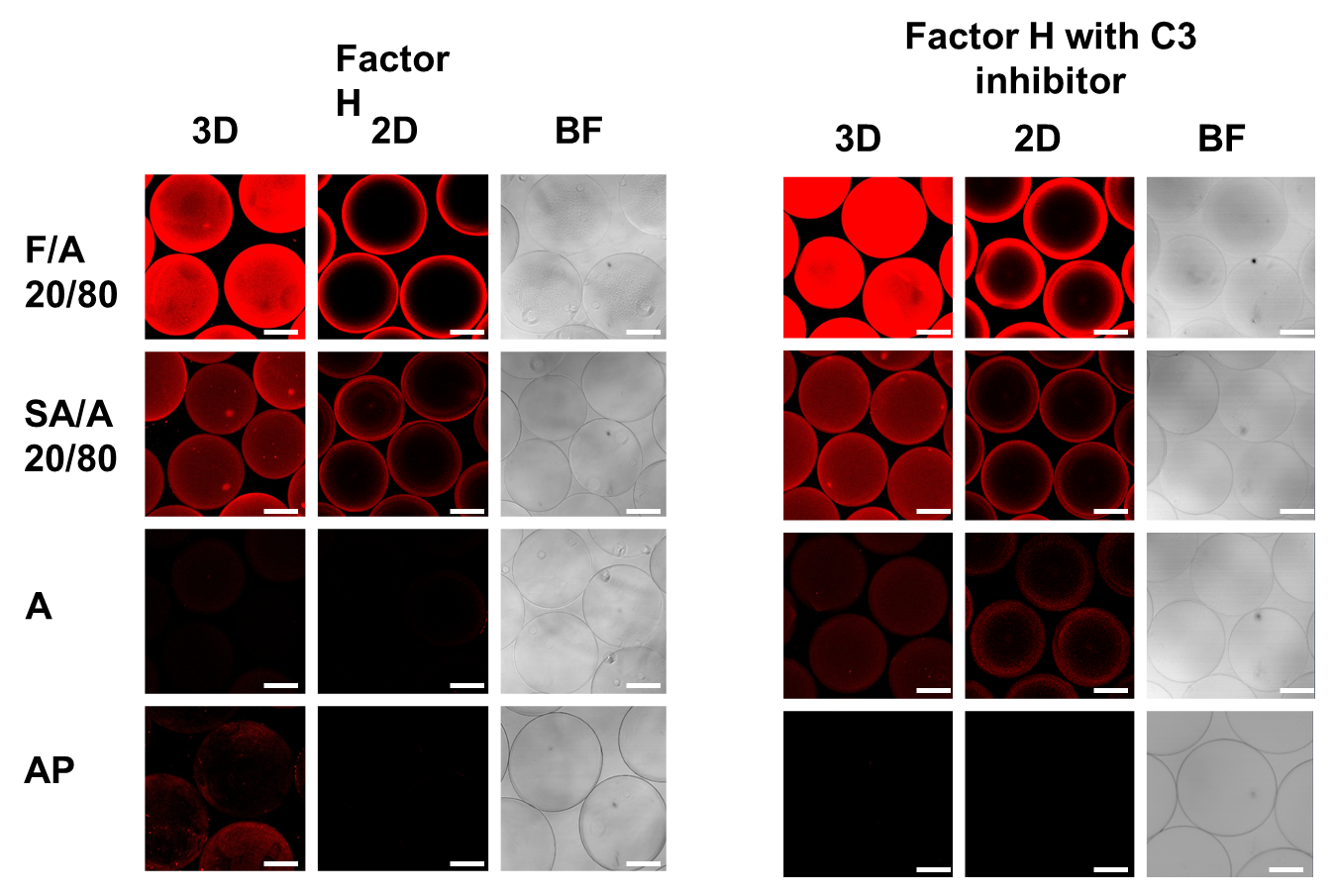


***Fig. S2****: Evaluation of complement and coagulation proteins and responses on/to microspheres. (A) Detection of factor H (CF633-stained - red) without C3 inhibitor (B) With Inhibitor (C) Coagulation protein; FXII (CF633-stained - red) (D) Plasminogen/Plasmin (CF633-stained - red) in human plasma by CLSM. CLSM images show antibody-stained microspheres after incubation (24 h) in hirudin-anticoagulated human plasma. Non-specific antibody binding was assessed. Captured images include brightfield (BF), equatorial (2D)-sections and (3D)-projections of z-stacked images. Scale bar = 200 µm*

Supplementary Figure 3 (Fig. S3)
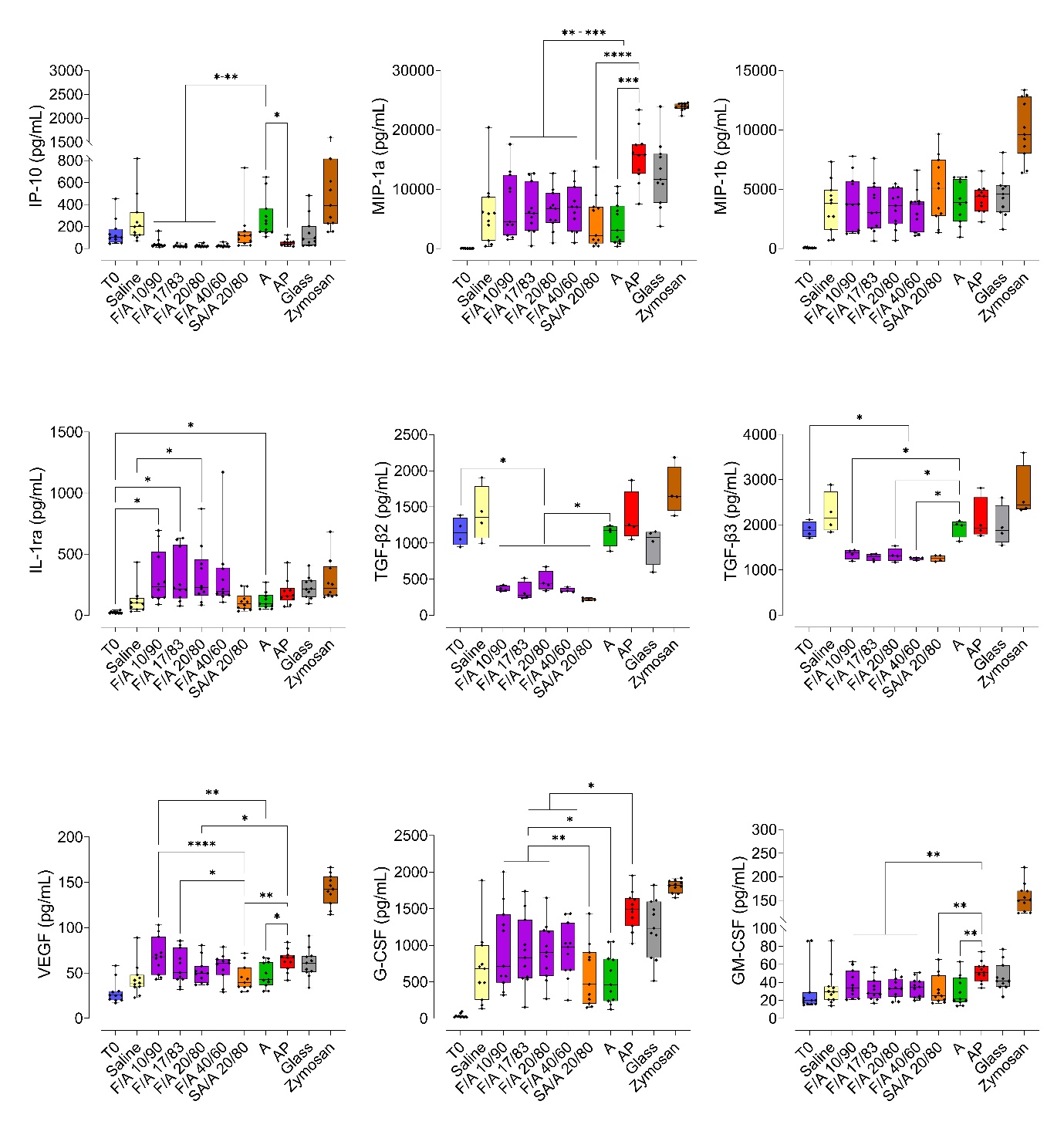


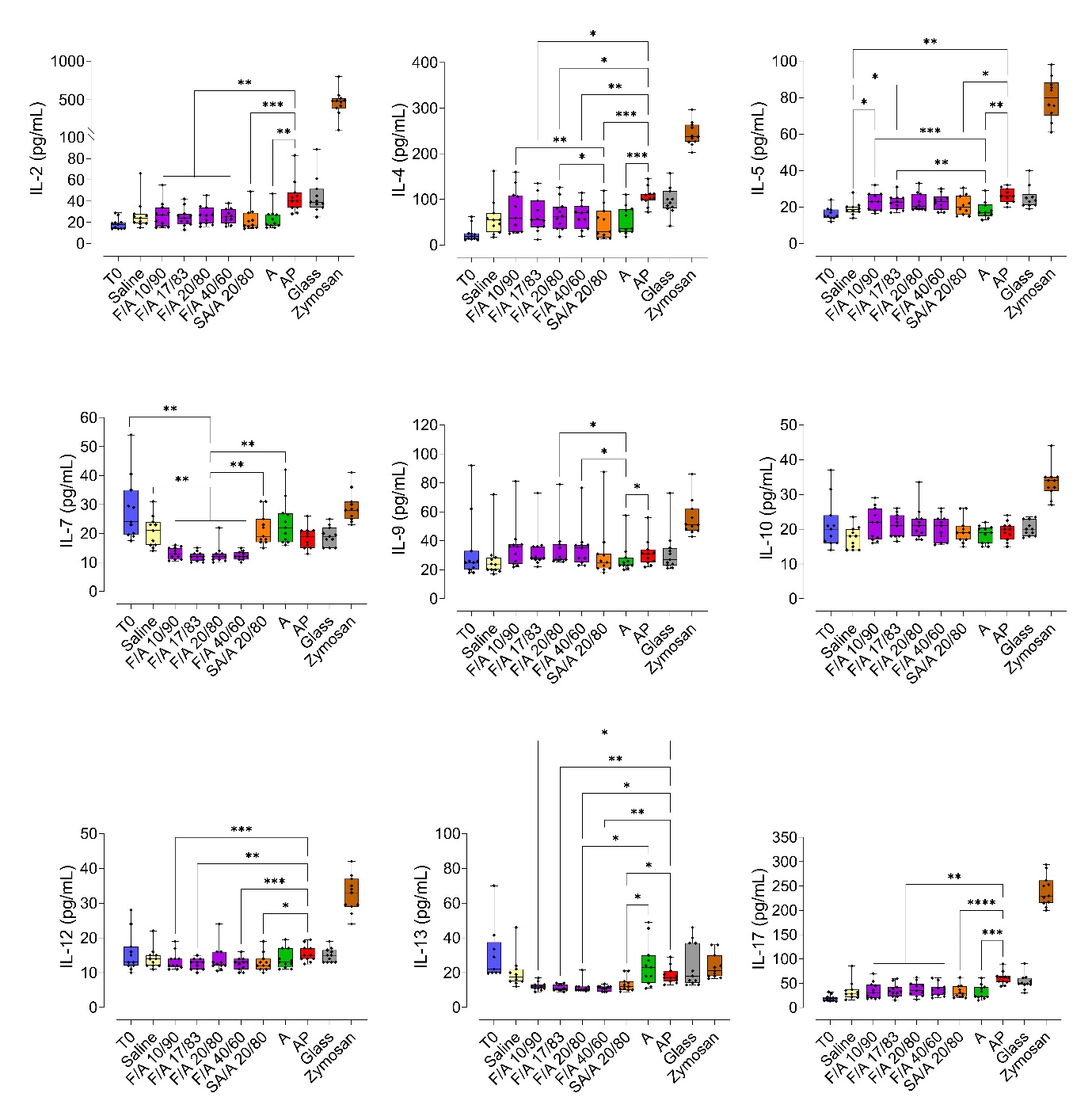


***Fig. S3:*** *Plasma concentrations (pg/mL) of inflammatory cytokines; IP-10, MIP-1α, MIP-1β, IL-1ra, TGF-β2, TGF-β3, VEGF, G-CSF, GM-CSF, IL-2, IL-4, IL-5, IL-7, IL-9, IL-10, IL-12, IL-13 and IL-17 following 4 h incubation of microspheres in whole blood anti-coagulated with hirudin (n = 11). Significant values are given as p ≤ 0.05 (*), p ≤ 0.01 (**), p ≤ 0.001 (***), p ≤ 0.0001 (****) compared to other bead types; SA/A, A and AP. T0 represents the experimental baseline. Data were analyzed by one-way ANOVA and Tukey HSD tests. Data that were not normally distributed were log-transformed before analyses.*

Supplementary Figure 4 (Fig. S4)

***Fig. S4:*** *Heatmap of the apolipoproteins identified on the surface of different microspheres. Protein abundances are coloured based on label-free quantification (LFQ) intensity values, indicated by colour scale bars (N= 4 technical replicates, 1 mouse)*
